# Unraveling the Relaxation Dynamics of Uracil: Insights from Time-Resolved X‑ray Photoelectron Spectroscopy

**DOI:** 10.1021/jacs.5c04874

**Published:** 2025-08-13

**Authors:** Davide Faccialà, Matteo Bonanomi, Bruno Nunes Cabral Tenorio, Lorenzo Avaldi, Paola Bolognesi, Carlo Callegari, Marcello Coreno, Sonia Coriani, Piero Decleva, Michele Devetta, Nađa Došlić, Alberto De Fanis, Michele Di Fraia, Fabiano Lever, Tommaso Mazza, Michael Meyer, Terry Mullins, Yevheniy Ovcharenko, Nitish Pal, Maria Novella Piancastelli, Robert Richter, Daniel E. Rivas, Marin Sapunar, Björn Senfftleben, Sergey Usenko, Caterina Vozzi, Markus Gühr, Kevin C. Prince, Oksana Plekan

**Affiliations:** † CNR, 96976 Istituto di Fotonica e Nanotecnologie, 20133 Milano, Italy; ‡ Dipartimento di Fisica, 274268 Politecnico di Milano, 20133 Milano, Italy; § Department of Chemistry, 5205 Technical University of Denmark, DK-2800 Kongens Lyngby, Denmark; ∥ Departamento de Química, Universidad Autónoma de Madrid, Madrid 28049, Spain; ⊥ CNR, Istituto di Struttura della Materia, 00133 Rome, Italy; # 18474 Elettra-Sincrotrone Trieste S.C.p.A., in Area Science Park, Basovizza 34149, Trieste, Italy; ∇ Dipartimento di Scienze Chimiche e Farmaceutiche, Universitá degli Studi di Trieste, I-34127 Trieste, Italy; ○ 54583 Institut Ruđer Bošković, Bijenička cesta 54, 10000 Zagreb, Croatia; ◆ 9315 European XFEL, Holzkoppel 4, 22869 Schenefeld, Germany; ¶ CNR, Istituto Officina dei Materiali, in Area Science Park, Basovizza, 34149 Trieste, Italy; ⋈ Institut für Physik und Astronomie, Universität Potsdam, 14476 Potsdam, Germany; ⧓ Deutsches Elektronen-Synchrotron DESY, Notkestraße 85, D-22607 Hamburg, Germany; ⧖ Laboratoire de Chimie Physique-Matiere et Rayonnement, LCPMR, CNRS, Sorbonne Université, Paris F-75005, France; ● Department of Physics and Astronomy, 8097 Uppsala University, Uppsala SE-75120, Sweden; ¤ Institut für Physikalische Chemie, Fachbereich Chemie, 28332 Universität Hamburg, Martin-Luther-King-Platz 6, 20146 Hamburg, Germany

## Abstract

We report a study of the electronic and nuclear relaxation dynamics of the photoexcited RNA base uracil in the gas phase using time-resolved core-level photoelectron spectroscopy together with high-level calculations. The dynamics was investigated by trajectory surface hopping calculations, and the core ionization energies were calculated for geometries sampled from these. The molecule was excited by a UV laser and dynamics probed on the oxygen, nitrogen, and carbon sites by core electron spectroscopy. We find that the main de-excitation channel of the initially excited S_2_(ππ*) state involves internal conversion to the S_1_(nπ*) state with a time constant of 17 ± 4 fs, while a portion of S_2_(ππ*) population returns directly to the ground state by internal conversion. We find no evidence that the S_1_(nπ*) state decays to the ground state; instead, it decays to triplet states with a time constant of 1.6 ± 0.4 ps. Oscillations of the S_1_(nπ*) state O 1s intensity as a function of time correlate with those of calculated C4=O8 and C5=C6 bond lengths, which undergo a sudden expansion following the initial π → π* excitation. Our calculations support our interpretation of the data and provide detailed insight into the relaxation processes of uracil.

## Graphical abstract

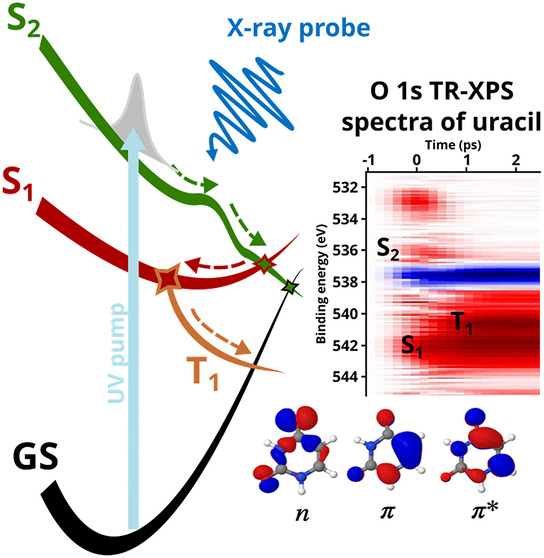

## Introduction

The newly available femtosecond short-wavelength pulses produced by synchrotrons, high harmonic generation (HHG), and free-electron laser (FEL) sources have enabled modern time-resolved experimental techniques to become an outstanding tool to probe ultrafast dynamical processes in nature.^1^ In particular, time-resolved photoelectron spectroscopy (TR-PES)^2,3^ has been recognized as an excellent method for monitoring photochemical reaction pathways in molecular systems, including the nonadiabatic dynamics taking place at conical intersections (CoIns).^4–7^

The vast majority of femtosecond TR-PES experiments with isolated molecules have been based on laboratory lasers at visible, ultraviolet, and extreme ultraviolet wavelengths,^8–10^ and have probed the valence levels. Fewer studies are based on core-level photoemission, which requires soft to hard X-rays. A key advantage of TR-PES with X-rays (TR-XPS) is the sensitivity of core electrons to their chemical environment.^11–17^ In addition, the technique is quantitative to a good approximation, and the signal intensity reflects the population of a given state, which is not the case with other methods, such as valence PES and X-ray absorption.

In this work, we applied TR-XPS with FELs to investigate the complex photoinduced dynamics of the isolated RNA base uracil (C_4_H_4_N_2_O_2_, Figure 1a). This pump–probe technique, exploiting a UV pump for the creation of excited electronic states and an X-ray probe for the ionization of the C, N, and O 1s core levels, provided insight into the ultrafast electronic relaxation process.

**Figure 1:**
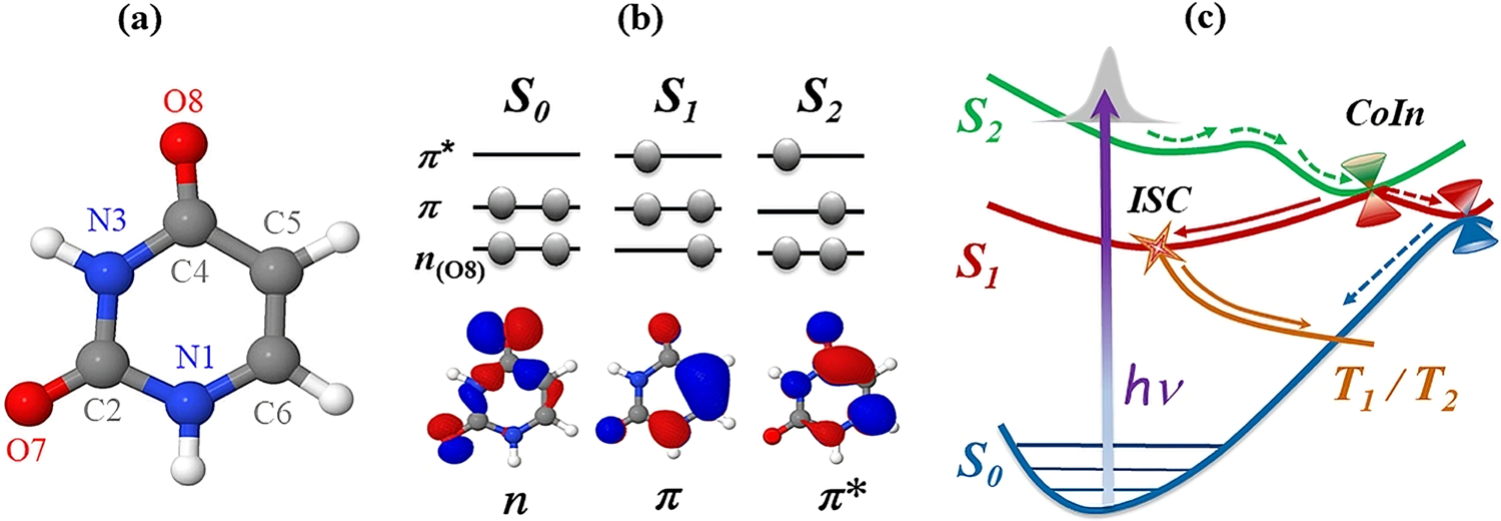
(a) Schematic structure of uracil with atom numbering. (b) Configurations of the ground S_0_ and two singlet excited S_1_, S_2_ states of uracil together with n, π, and π* molecular orbitals. (c) Sketch depicting uracil relaxation after UV excitation, with adiabatic potential energy curves (PEC) of the excited singlet (green, red) and triplet (orange) valence states along with the ground state (GS) (blue). The conical intersections (CoIn) between S_2_/S_1_ and S_1_/S_0_ PEC together with S_1_/T_1,2_ intersystem crossing (ISC) are indicated. Experimentally observed direct and indirect relaxation pathways are specified by dashed and solid arrows, respectively.

Several experimental and theoretical studies have focused on the time-resolved X-ray absorption (TR-XAS) of organic chromophores, where core-level absorption, which in some respects is experimentally easier than photoelectron spectroscopy, is used as the probe of photoinduced dynamics, see, e.g., refs 18–24 . A few theoretical studies of core phototoelectron spectra of UV-excited DNA and RNA bases have also appeared recently.^25–27^ Mukamel and coauthors have proposed a new transient redistribution of ultrafast electronic coherences in attosecond Raman signals (TRUECARS) technique^28^ and have simulated the high temporal resolution TR-PES signal in order to monitor the photorelaxation of uracil passing through a CoIn.^29^ However, studies of TR-XPS of gaseous nucleobases remain rather limited.^15,16,25–27^

A combined experimental and theoretical study of the ground state (S_0_ or GS) XPS spectra of uracil has been previously published.^30^ In addition, other calculations have been reported.^31,32^

Figure 1b displays the highest occupied molecular orbital (HOMO), denoted π, and the nonbonding HOMO–1 orbital, denoted n, along with the lowest unoccupied molecular orbital (LUMO), π*. At the Franck–Condon (FC) geometry, the lowest energy excited state S_1_ has nπ* character and arises from the excitation of a lone pair electron initially localized on the O8 atom (see Figure 1a,b) to the delocalized π* orbital. The S_1_ state is optically dark, i.e., not accessible from the S_0_ state via one-photon absorption.^33^ The second excited state S_2_ is optically bright and corresponds to a ππ* transition, with an electron promoted from the bonding π orbital (localized mostly on the C5=C6 and C4=O8 bonds) to the corresponding antibonding π* orbital (see Figure 1b).

There is a significant difference in charge distribution between the ππ* and nπ* states, particularly at the O8 oxygen atom (see Figure 1b for the orbital charge densities, and Figure S1, Section S1, Supporting Information (SI), for the charge density differences and further details). Recently, calculations by Vidal et al.^25,26^ have predicted clear differences between the theoretical O 1s XPS spectra of the two excited states, and the ground state, associated with differing charge distributions. In particular, a chemical shift of about 4 eV from GS was predicted for S_1_ ionization of O8. For the N 1s and C 1s core electrons, the changes in local charge density are smaller, so that the shifts were less pronounced, but still expected to be observable in TR-XPS spectra.^25,26^

The photodynamics of uracil has been investigated both experimentally and theoretically, see ref 34 for a recent summary. These studies have found that upon excitation to the S_2_(ππ*) state, the relaxation of uracil back to S_0_ takes place nonradiatively, along two, or possibly three, competing pathways depicted in Figure 1c. This includes a direct relaxation along the ππ* state, in which at the S_2_/S_1_ CoIn part of the S_1_ population remains in the ππ* state, and reaches the CoIn with the S_0_ state (Figure 1c, green/red/blue dashed arrows).^34–38^

In the indirect pathway, the remaining part of the population switches from the ππ* to the nπ* state at the S_2_/S_1_ CoIn (Figure 1c, red solid arrow). In the S_1_(nπ*) state, the population remains trapped for several picoseconds and eventually decays by intersystem crossing (ISC) to the triplet manifold (T_1_/T_2_) (Figure 1c, orange solid arrow).^36,39,40^ Some early works have also suggested trapping of the population in the S_2_(ππ*) state and decay via ring opening.^41^ Within this general framework, the time constants associated with specific relaxation steps are still under debate.^34–47^

This paper presents a detailed study of the core-level photoelectron spectra of gaseous uracil as a function of the pump–probe delay, together with preliminary data for N and C 1s, to monitor the relaxation pathway of uracil after UV excitation. We exploit the fact that core binding energies (BEs) provide quantitative local chemical information about the probed atoms in a given system. To obtain a detailed insight into both the mechanism and time scale of ultrafast processes in isolated uracil, we have coupled extensive nonadiabatic dynamics simulations with high-level multireference calculations of TR-XPS signals. We determine time constants for key processes and observe the effects of nuclear dynamics on the XPS signals.

## Methods

### Theoretical Section

The computational protocol for simulating time-resolved XPS spectra is based on a trajectory surface hopping (SH) description of the excited state dynamics of uracil (see ref 38) and the calculation of the core ionization energies and intensities from geometries sampled from SH trajectories. The accuracy of this protocol has been illustrated in recent publications.^48,49^ For more details of the nonadiabatic dynamics simulations, see Sections S1–S3, Supporting Information.

Time-resolved XPS spectra of uracil were computed at early pump–probe delay times using 48 SH trajectories (see Table S1, Section S1, Supporting Information), and employing the RASPT2 method combined with the aug-cc-pVDZ basis set. The active space utilized in the restricted active space self-consistent field (RASSCF) calculations is divided into three segments: RAS1, RAS2, and RAS3.^50,51^ RAS1 comprises the relevant core orbitals, RAS2 includes seven valence-occupied orbitals, and RAS3 is formed by two π* orbitals, each capable of accommodating a maximum of two electrons. The third highest-lying π* orbital, which is not directly involved in the physical description of the excited states of interest, was excluded in order to reduce the computational costs.

Core–hole states were calculated by enforcing a single hole in RAS1 using the HEXS projection technique,^52^ available in OpenMolcas.^53^ RASSCF orbitals of initial valence-excited and final core ionized states were obtained by state averaging over 10 and 20 states, respectively. The state-averaged active orbitals, computed at the equilibrium geometry of the ground state, are schematically represented in Figure S2 (see Section S1, Supporting Information). To incorporate dynamical correlation effects, the extended multistate restricted active space perturbation theory of the second order (XMS-RASPT2) approach^54^ was employed in the reference space. An imaginary level shift of 0.35*E*_h_ was applied to avoid intruder-state singularities.

The simulated XPS spectra for both the ground state and valence excited states of uracil were generated through convolution of the computed ionization energies and the squared norms of Dyson orbitals, that is, pole strengths, employing a Lorentzian function of typically 0.4 eV (full width at half-maximum (fwhm)). Dyson orbitals were computed following the procedure outlined in ref 55, utilizing the RASSI^56^ module of OpenMolcas^53^ with the RASPT2 energies and the perturbatively modified (mixed) RASSCF transition densities.^57^ To assess the quality of the XPS spectra derived from the squared norms of the Dyson orbitals, we also calculated cross sections using an explicit description of the electronic continuum obtained at the density functional theory (DFT) level with a linear combination of atomic orbitals (LCAO) B-spline basis, employing the Tiresia code.^58^ A detailed comparison is provided in Figure S3 (see Section S2, Supporting Information).

### Experimental Section

The experiment was performed at the Small Quantum Systems (SQS) instrument located at the European X-ray Free-Electron Laser (XFEL) facility.^59–61^ Isolated uracil molecules were irradiated by femtosecond soft X-ray pulses at the atomic-like quantum systems (AQS) experimental station. Uracil was evaporated by a capillary oven at a temperature of 433 K into an ultrahigh-vacuum chamber, with a sample density of about 10^12^ cm^–3^ in the interaction region.^62^

The SASE3 soft X-ray undulator was tuned to provide X-ray pulses centered at a photon energy of 600 eV. This photon energy was sufficient to ionize all three O, N, and C 1s core levels of uracil (see Section S12, Supporting Information, for the C 1s spectra). The XFEL beam pulses, with a duration of about 30 fs, were focused to a diameter of approximately 100 μm (fwhm) in order to minimize nonlinear effects. The SQS instrument monochromator^63^ was used to reduce the initial FEL bandwidth to 0.136 eV (fwhm), resulting in a pulse energy of 0.21 μJ on the target (see Figure S5, Section S4, Supporting Information)

Uracil molecules were excited into a bright S_2_ electronic state by UV pump pulses at a wavelength of 264 nm (4.70 eV). The duration of these pulses was measured to be about 75 fs, and the focus diameter was 150 μm (fwhm). The pulse energy was around 5 μJ, a value chosen to curb excessive pumping and avoid two-photon ionization by the optical laser (see Figure S8, Section S4, Supporting Information).

A delay stage was used for variation of the temporal interval between the UV pulses and the X-ray pulses. A nominal step size of 20 fs was used in the range from −200 to +500 fs, where a negative delay indicates that the X-ray pulse arrived before the UV pulse. In addition, spectra were also acquired at fixed delays of +1 ps, +10 ps, +100 ps, and +1 ns. The nominal delays were corrected by means of the pulse arrival-time monitor^64^ and rebinned. A bin size of 18 fs was chosen so that all bins have similar statistics.

Photoelectron spectra as a function of the time delay between the UV and soft X-ray pulses were recorded with the magnetic bottle electron spectrometer (MBES) of the experimental station. The X-ray photons were linearly polarized perpendicular to the axis of the magnetic bottle spectrometer and the UV polarization. In order to enhance the energy resolution of the MBES spectrometer, 1s electrons from oxygen, nitrogen, and carbon were retarded to an ∼25 eV final kinetic energy using appropriate retardation voltages.

Photoelectron spectra of the ground and photoexcited states of uracil were measured in consecutive shots by pulsing the UV laser at half the repetition frequency of the XFEL: with the UV laser off, the ground state was measured; with the UV laser on, the excited state plus a fraction of ground state molecules were measured. If the fraction of excited molecules is denoted *f*, then the fraction of ground state molecules in this case is 1 – *f*. The raw data were analyzed by using two approaches. First, the UV-off spectra were subtracted directly from the UV-on spectra, yielding difference spectra in which the excited state features were positive and the ground state features were negative; that is, they appeared as depletion of the signal. Second, we performed a scaled subtraction for the fraction of ground state molecules in the UV-on spectrum,^65^ thereby eliminating negative features. The fraction of excited molecules was estimated to be *f* = 0.09 using the procedure described in the Supporting Information (see Section S5).

## Results

### Theoretical Predictions and Dynamical Model

Our surface hopping calculations predict that the S_2_ state decays on an ultrafast time scale. At S_2_/S_1_ CoIn, the population (trajectories) bifurcates. One portion of the trajectories (36%) continues to evolve on the diabatic ππ* state, which drops below the nπ* state in energy. Consequently, this state becomes the first excited state and is denoted as S_1_(ππ*). It subsequently crosses with the ground state, resulting in the relaxation pathway: S_2_(ππ*) → S_1_(ππ*) → S_0_ (see Figure S14, Section S7, Supporting Information). This fraction of the population gives rise to the hot ground state (HGS), as each molecule contains the energy of a UV photon as vibrational energy. The above calculated quantum yield is in reasonably good agreement with the experimental result of ref 66, in which it was found that 45% of the initially excited state returns to the ground state.

The other portion of the population switches to the nπ* state (64%) at the S_2_/S_1_ CoIn and continues along the gradient on this surface, which in this region corresponds to the first adiabatic S_1_(nπ*) state. This state does not undergo internal conversion (IC) to the ground state as the S_1_(nπ*)/S_0_ CoIn lies too high in energy. Instead, due to the strong spin–orbit coupling and the small energy gap between the S_1_(nπ*) state and the two lowest triplet states, it undergoes intersystem crossing to the triplet manifold, leading to the alternative pathway: S_2_(ππ*) → S_1_(nπ*) → T_1_/T_2_.

### Oxygen K-Edge

The calculated and experimental time-resolved O 1s spectra of valence-excited uracil are shown in Figure 2a,b, respectively. Table 1 summarizes the theoretical O 1s binding energies (BEs) for the S_0_, S_1_, and S_2_ states at the equilibrium geometry of the ground state and their corresponding ionization characters.

**Figure 2:**
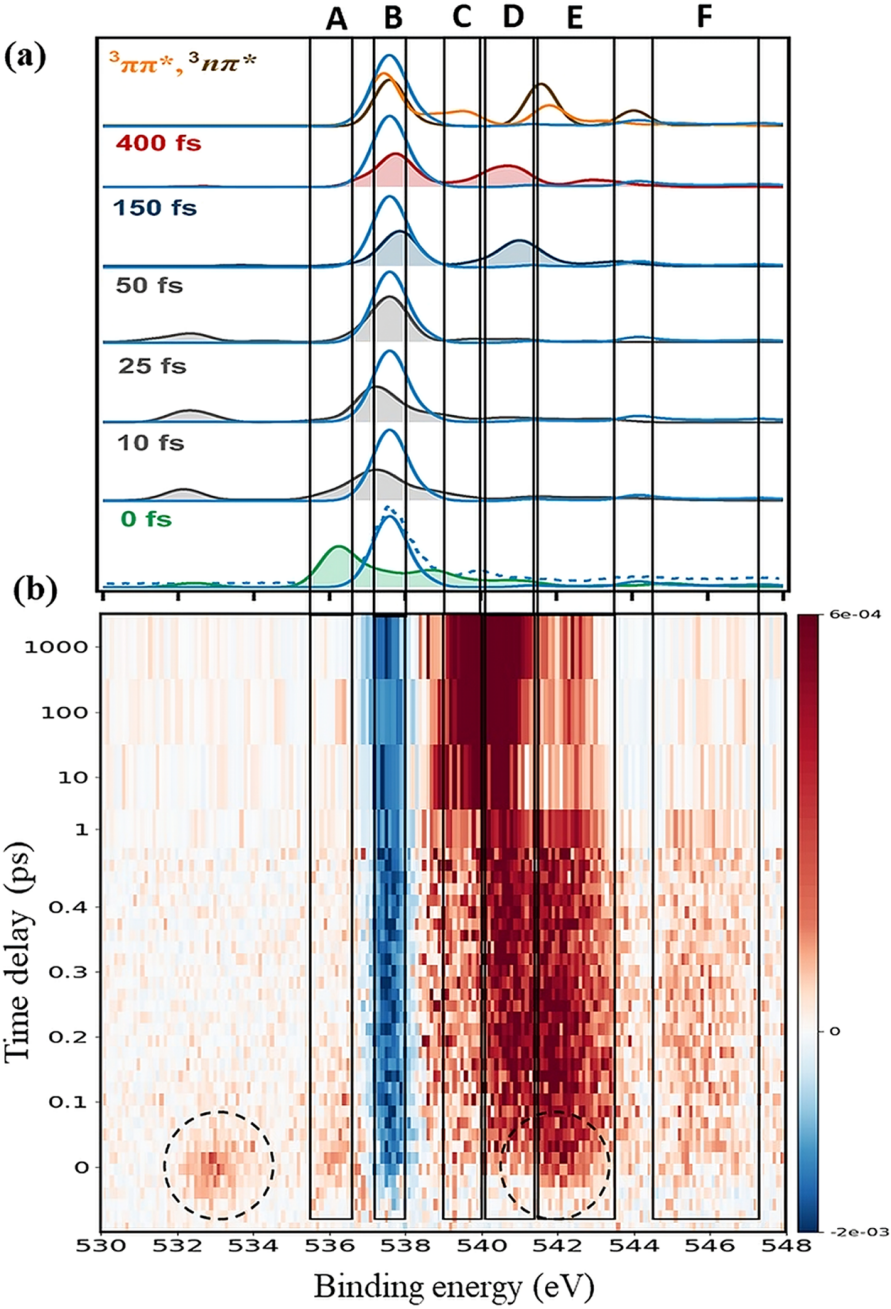
(a) Calculated O 1s spectra of uracil in the ground (blue solid line) and singly excited states (shaded areas: ^1^ππ* (green/gray) and ^1^nπ* (blue/red)) at selected time delays (see Table S1, Section S1, Supporting Information), and triplet states ^3^ππ* (orange line) and ^3^nπ* (brown line). Theoretical spectra are shifted by 2.4 eV to a lower binding energy. The blue dashed line represents the experimental GS spectrum. (b) Two-dimensional false color map of the O 1s difference spectra (UV-on minus UV-off) as a function of binding energy and the time delay (red: positive signal; blue: negative signal). Black dashed circles indicate sidebands (SBs). Energy ranges (eV): (A) 535.9–536.6; (B) 537.2–538.0; (C) 539.0–540.0; (D) 540.1–541.5; (E) 541.6–543.5; (F) 544.5–547.5.

**Table 1:** Theoretical O 1s Ionization Energies (in eV) and Orbital Character Computed at the RASPT2/aug-cc-pVDZ Level in the FC Geometry for Three Initial States^a^

initial state	BE (eV)	ionization character
GS	539.95	O8 1s^–1^
	540.05	O7 1s^–1^
	539.86	O7 1s^–1^nπ*
S_1_(nπ*)	544.02	O8 1s^–1^nπ* (L)
	546.40	O8 1s^–1^nπ* (H)
	534.20	O8 1s^–1^(shake-down)
S_2_(ππ*)	538.26	O8 1s^–1^ππ*
	539.17	O7 1s^–1^ππ* (L)
	540.41	O7 1s^–1^ππ* (H)

a Due to the unpaired electrons in the S_1_ and S_2_ states, primary core ionization yields final doublet states with 3 unpaired electrons, which are split into several spin multiplets; labels L and H indicate lower and higher energy states. For a discussion of spin coupling in the case of X-ray absorption of doublet molecular cations, see, e.g., refs 68–71 .

The computed energies presented here for the two O 1s electrons in the GS are 539.95 and 540.05 eV, about 2.4 eV higher than the experimental values. Such discrepancies are common and are due to an incomplete description of core relaxation effects, electron correlation, and relativistic effects. All theoretical spectra in Figure 2a have been rigidly shifted by −2.4 eV to align them with the experimental results. For the S_2_ state, the calculated binding energy of O8 with final state configuration O8 1s^–1^ππ* was 538.3 eV. The weak feature computed near 534 eV is assigned to a shake-down transition of the S_2_ state.^67^ Note that the theoretical shake-down energy does not match exactly the experimental excitation energy. This is because in the adopted computational approach the valence excitation energy of the S_2_ state (5.75 eV) overestimates (in the vertical approximation) the energy of the pump (4.70 eV). For the S_1_ state, the BE of O8 1s with the final state configuration O8 1s^–1^nπ* was calculated to be 4 eV higher than the GS peak (see Table 1), consistent with the previous prediction of Vidal et al.^25,26^ In contrast to the calculated BEs of the O8 atom, those of O7 are shifted only slightly from the ground state BE in the S_2_(ππ*) or S_1_(nπ*) state (see Table 1).

The experimental and theoretical O 1s spectra presented in Figure 2 were divided into six BE ranges (A–F) in which signal intensity variations are evident. As noted above, data were acquired in two different time delay intervals (Figure 2). The first range (from −100 to +450 fs) maps ultrafast dynamical changes, while the second range (from 1 ps to 1 ns) follows the long-lived excited states.

Difference spectra are plotted as a false color, two-dimensional map in Figure 2b, as a function of binding energy and time delay. This representation shows the valence excited states as positive features, while negative features are due to the depletion of the ground state. The two experimental features at 532.9 and 542.3 eV observed around zero time delay (see Figure 2b, dashed circles) are due to the lower and upper sidebands (SBs) resulting from the simultaneous absorption of a soft X-ray photon and absorption (or emission) of a UV photon. The maximum intensity of the low-energy sideband is equal to about 0.23 times the asymptotic depletion of the main peak. Since the SB signal occurs only when the pump and probe pulses overlap temporally, it serves as a convenient monitor for the precise measurement of their cross-correlation. Note that the high-energy sideband overlaps the structure of interest in region E; the exact onset of the structure is extracted by fitting (see Figure S10, Section S6, Supporting Information), and assuming the same line profile as the low-energy sideband. From the fit, it is clear that the sidebands appear for a very short time before the new intensity in range E.

The weak intensity observed around 536 eV in range A is assigned to excitation of the S_2_(ππ*) state (see Table 1 and Figure 2a, panel at 0 fs), and its disappearance is ascribed to rapid structural changes. Experimentally the ultrafast decay of the S_2_ state signal in range A coincides with the appearance of signals in ranges C, D, and F. Based on the computed ionization energies (Table 1) and calculated spectra (Figure 2a, panel at 150 fs), the intensities in ranges C–E are attributed to the nπ* state, which is populated via S_2_ → S_1_ internal conversion. In the simulations, the S_2_(ππ*) signal shifts from range A to B within approximately 25 fs, driven by rapid geometric distortions of the molecule after photoexcitation (see Figure S14, Supporting Information). However, the appearance of signals in ranges C, D, and E occurs on a slower time scale in the simulations than in the experiment, indicating that while the calculations correctly describe the decay mechanism of the S_2_(ππ*) state, they overestimate its lifetime. Furthermore, a recent TR-PES experimental study with unprecedented time resolution has reported for the S_2_(ππ*) state of gaseous uracil an ultrafast deactivation lifetime ≈17 fs.^66^

According to our calculations (see Table 1), the S_1_ state feature exhibits an asymmetric doublet structure with an energy difference of approximately 2.4 eV (see Figure S3, Section S2, and Supporting Information). This is attributed to the spin coupling of the three unpaired electrons: one in the n orbital, one in the π* orbital, and one in the 1s orbital of O8, resulting in a final core ionized state of doublet spin multiplicity.

In range B (537.2–538 eV), the depletion of the ground state is clearly visible as a single negative peak due to ionization in the region of the O7 and the O8 binding energies. Our calculations indicate that the depletion is due to a large energy shift of O8 in the excited states, while the ionization energy of O7 does not change significantly (see Table 1). This implies that the integral of the depletion signal is equal to the integral of the O8 1s signal of the excited state. Our calculations suggest that, a portion of the excited state population returns to the ground state, giving rise to a vibrationally hot ground state (see discussion below and Section S3, Supporting Information).

In the present O 1s spectra, the core ionization of the excited states does not correspond simply to a single electron ionization from a closed-shell configuration because there are two singly occupied valence levels, leading to a final state with three unpaired electrons. This facilitates shake processes, that is, transitions to 2-hole 1-particle (2h1p) states.^67^ The observed intensity between 544.5 and 547.5 eV (Figure 2b, range F) is attributed to a shakeup process of the molecules in the S_1_ state, terminating in less than 10 ps, which aligns with the gradual depopulation of the dark S_1_ state of uracil. For example, in Figure S15 (see Section S8, Supporting Information), the shakeup signal appears above the 546 eV region in the spectra for a selected SH trajectory which undergoes IC to the S_1_(nπ*) state. Hence, the UV excitation of uracil made it possible to experimentally observe final states that are only weakly excited in XPS of the ground state but have much higher relative intensities in the current O 1s TR-XPS spectra.

### Nitrogen K-Edge

The theoretical and experimental N 1s photoelectron spectra of photoexcited uracil are presented in Figure 3a,b, respectively. The experimental spectra were collected for a short range only, from −150 to +450 fs, with limited statistics, but are still sufficient to follow the charge dynamics around the nitrogen atoms. As in the O 1s spectra, the ground state is depleted on excitation, but in contrast to the O 1s spectra, the depletion partially recovers after about 50 fs. Positive and negative sidebands are also observed, Figure 3b, with black circles. The ratio of the low-energy sideband intensity to the asymptotic attenuation is 1.3, considerably more than in the case of oxygen.

**Figure 3:**
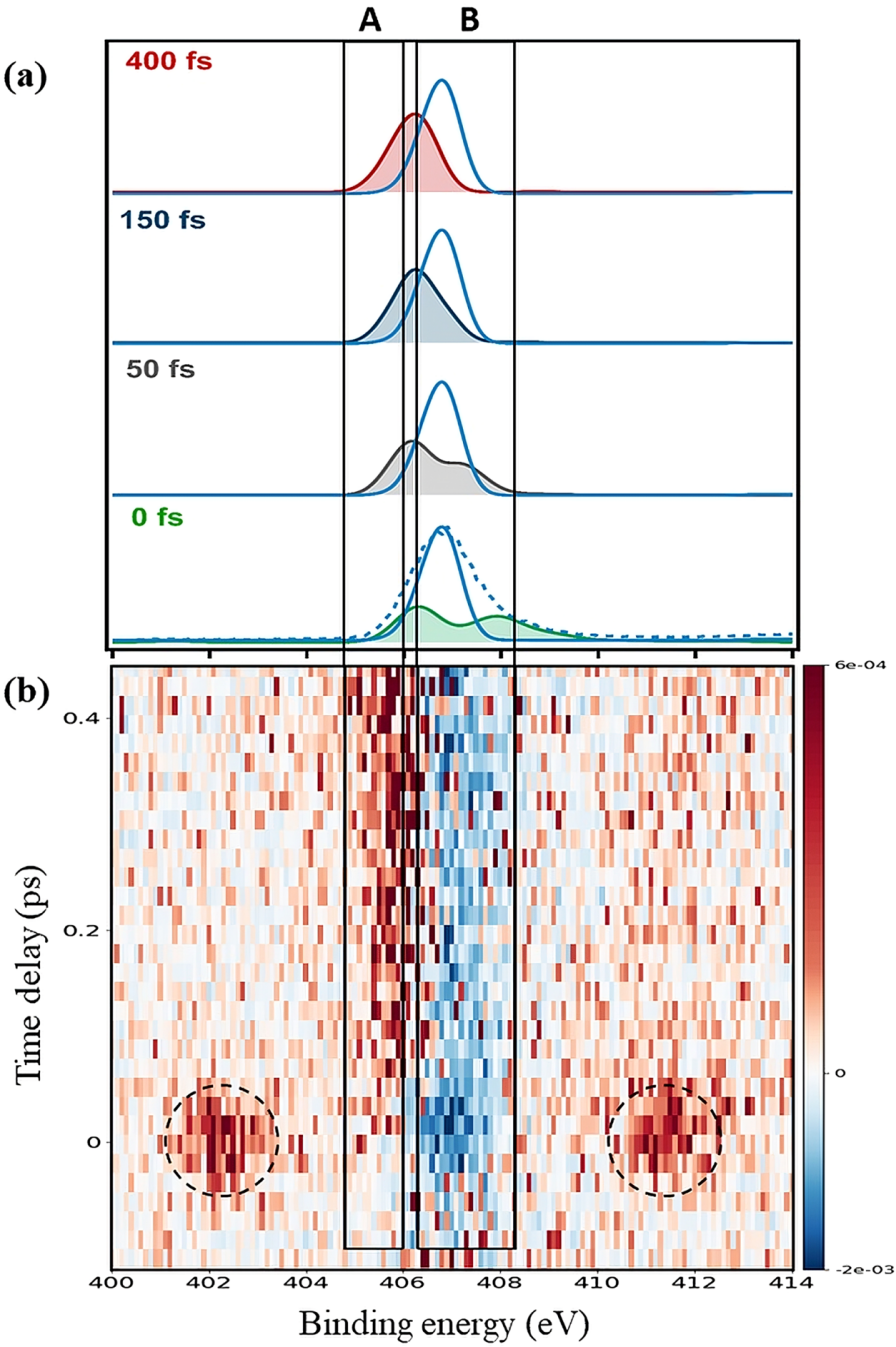
(a) Calculated N 1s spectra of uracil in the ground (blue solid line) and singly excited states (shaded areas: ^1^ππ* (green/gray) and ^1^nπ* (blue/red)) at selected time delays (see Table S1, Section S1, Supporting Information). Theoretical spectra are shifted by 2.1 eV to a lower binding energy. The blue dashed line represents the experimentally measured GS spectrum. (b) Two-dimensional false color map of the N 1s difference spectra (UV-on minus UV-off) as a function of binding energy and time delay (red: positive signal, blue: negative signal). Black dashed circles indicate sidebands. Energy ranges (eV): (A) 404.8–406.0 and (B) 406.3–408.3.

The sideband intensity scales with the kinetic energy of the electron, and the kinetic energy of the N 1s electron is higher than that of the O 1s. Thus, the recovery of the depletion signal when the sideband channel closes is more evident for N 1s. To check this hypothesis, we show in Figure S16 (see Section S9, SI) that by adding the intensity of the sidebands to the depletion signal, the trend of the depletion is qualitatively similar to that of oxygen.

The calculated N 1s BEs for the S_0_, S_1_, and S_2_ states at the equilibrium geometry of the ground state, and their corresponding ionization characters, are summarized in Table 2. The spectrum of the S_2_(ππ*) state at zero time delay consists of two peaks, one at slightly lower binding energy than the ground state due to N3 ionization (408.38 eV), and the other peak due to N1 ionization (410.24 eV). However, our experimental spectra do not show the latter peak, which may be due to insufficient instrumental resolution (see Figure 3a, blue dashed line). For longer time delays of 150 and 400 fs, theory predicts that the spectral signature of the S_1_(nπ*) state consists of a single, broadened peak at slightly lower binding energy than the ground state. This is indeed visible in Figure 3b as an intensity increase in range A, and depletion in range B.

**Table 2:** Theoretical N 1s Ionization Energies (in eV) and Orbital Character Computed at the RASPT2/aug-cc-pVDZ Level and at the FC Geometry for Three Initial States

initial state	BE (eV)	ionization character
GS	408.77	N3 1s^–1^
	409.10	N1 1s^–1^
S_1_(nπ*)	408.58	N3 1s^–1^nπ*
	408.93	N1 1s^–1^nπ*
S_2_(ππ*)	408.38	N3 1s^–1^ππ*
	410.24	N1 1s^–1^ππ*

## Discussion

We begin by discussing the time constants for the decay of the S_2_(ππ*) and S_1_(nπ*) states, as determined from the experiment, and then relate them to the decay mechanisms revealed by the simulations. As mentioned in the Introduction, S_2_ and S_1_ are often used to denote the diabatic ππ* and nπ* states, though this is not strictly correct: S_1_ refers to the first adiabatic excited state, and S_2_ to the second adiabatic excited state. We use these labels in combination with the diabatic notations ππ* and nπ* for consistency with earlier work.

The photoelectron intensities integrated over the marked regions in Figure 2b were plotted as a function of time. A global fitting analysis was applied to extract the time constants, with the results shown in Figure 4 (see Section S6, Supporting Information, for more details). The lower binding energy sideband (Figure 4, range SB) was fitted with a Gaussian profile and yielded a cross-correlation time of 80.5 ± 4 fs.

**Figure 4:**
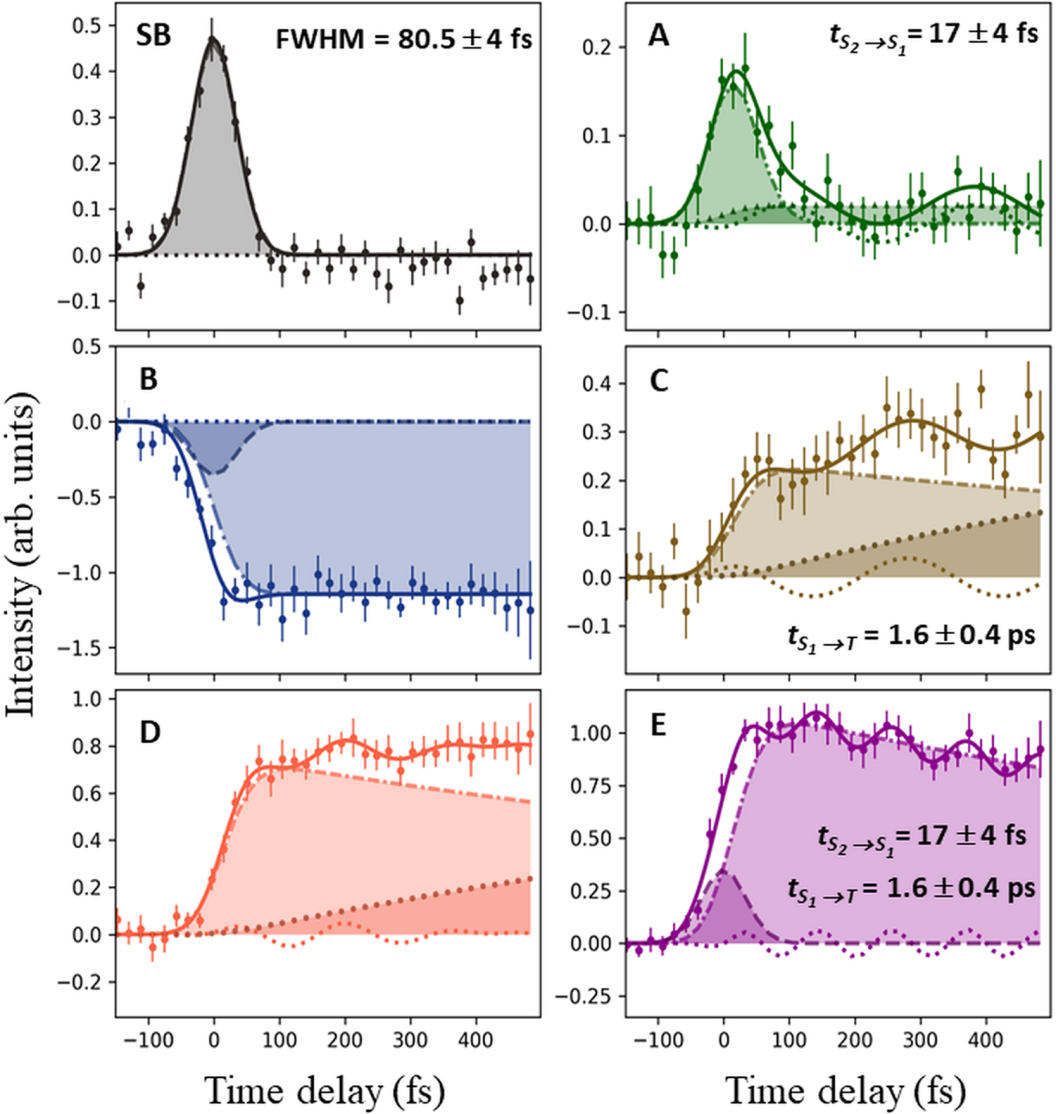
O 1s photoelectron intensity integrated over BE regions SB, and A to E (see Figure 2b) as a function of time delay. The decay time constants are extracted from the global fit (Section S6, Supporting Information) of the O 1s data. Shaded areas represent the population dynamics of the corresponding states identified by the fit. The oscillations (square dots, line) in the O 1s signal intensities for the ranges A, C, D, and E are also shown.

The applied model assumes a Gaussian excitation function, followed by an exponential decay describing the conversion of S_2_(ππ*) to S_1_(nπ*). At later times, an exponential decay describes the intersystem crossing of the S_1_(nπ*) state to the two lowest triplet T_1_/T_2_ states. A portion of the population of the initially excited S_2_(ππ*) state undergoes direct deactivation to the electronic ground state (see dashed arrows in Figure 1c), leading to the formation of the HGS. However, our theoretical simulations indicate that the time the trajectories spend in the S_1_(ππ*) state after passing through the S_2_/S_1_ CoIn (dashed red arrow in Figure 1c) is relatively short—approximately 30 ± 11 fs—compared to the computed lifetime of the ππ* state, which is 161 ± 40 fs. Considering that the computations overestimate the lifetime of the ππ* state, we conclude that the residence time in the S_1_(ππ*) state falls below the experimental time resolution. Consequently, as we are not able to observe this contribution separated from other signals, the transition to HGS was assumed to be instantaneous.

Within this model, the exponential time constant representing the conversion from the S_2_ to the S_1_ state has an average value of 17 ± 4 fs (see Figure 4, range A), much shorter than in most previous measurements.^40,45,72–74^ Despite this time constant being smaller than the response function of the instrument, we are able to capture it with high precision due to the high signal-to-noise level of S_1_, and the good spectral separation between the signals representing the S_2_ and S_1_ populations. Our value is model-dependent, since the S_2_ is assumed to be entirely transferred to the S_1_ state, which means the S_2_ decay equals the S_1_ rise time, while the HGS population is introduced to account only for the nonzero signal in the range A at higher delays. As such, its accuracy depends on the correctness of the model. However, SH calculations predict an average decay time for the diabatic ππ* state (S_2_ + S_1_) that is less than 20% longer than the rise time of the S_1_(nπ*) state. This difference is smaller than our experimental resolution, and it supports the reasonableness of the adopted approach. Moreover, we mention that the above time constant agrees exceptionally well with the value of 17 ± 1 fs reported by Miura et al. for the S_2_ decay.^66^

The calculated O 1s spectra at 0, 10, 25, and 50 fs show that as the molecule evolves on the S_2_(ππ*) surface, the signal at 536.0 eV gradually shifts toward higher binding energies. By 50 fs, the computed signal becomes indistinguishable from that of the ground state, even though most trajectories still reside in the ππ* state (see Figure 2a). This time scale is longer than the ”best estimate” of 12.5 fs for the decay of S_2_ obtained by Matsika and co-workers using XMS-CASPT2-based surface hopping simulations.^37^ In our SH simulations based on SCS-ADC(2), the S_2_(ππ*)/S_1_(nπ*) CoIn is less accessible, probably due to a tiny barrier in the S_2_(ππ*) state. This reduced accessibility causes our method to overestimate the decay time constant. Nevertheless, the underlying direct relaxation mechanism, S_2_(ππ*) → S_1_(ππ*) → S_0_, is the same. Finally, the direct pathway of S_2_(ππ*) decay (see Figure S14, Section S7, Supporting Information) leads to a population of vibrationally excited molecules (or HGS) in the electronic ground state via an ethylenic-type seam of CoIns, which involves a twist around the C5=C6 bond.^34,36–38,47,75^

To identify the spectral signature of the vibrationally excited molecules, we calculated the O 1s ground state spectra for the trajectories that returned to the S_0_ state, synchronizing them to start from the respective S_1_(ππ*)/S_0_ CoIns at *t* = 0. Our calculations show that the signal appears slightly above the GS energy, varies with time delay up to 1 ps, and is asymmetric (see Figure S4, Section S3, Supporting Information). Indeed, the shift in the O 1s ground state peak toward higher binding energies is consistent with the observed broadening of the main peak and the increased intensity between 538 and 539 eV (see Figure 2b), which we attribute to the HGS (see discussion below, and Section S6, Supporting Information).

For the S_1_(nπ*) state, we extracted an experimental decay constant of 1.6 ± 0.4 ps (see Figure 4, range E). The trapping of the population in the S_1_(nπ*) state with the indicated time constant is in agreement with several previous experimental studies.^34,40,66,72,73^ However, unlike Miura et al.,^66^ we find no evidence that the system decays to the ground state after reaching the nπ* state, as the ground state depletion signal does not recover in the measured O 1s spectra, even at times up to 1 ns (see Figure 2b). Instead, we find the S_1_ state to be the doorway to the triplet states (see Figure 4, range C–D).^36,39,42–44^ To test this hypothesis, we computed the spectra for the two lowest triplet states, ^3^nπ* and ^3^ππ*, at their respective minimum energy geometries. We note that the ordering of these states is geometry-dependent and that each can stabilize as the lowest triplet state (T_1_). Based on these computations (see Figure 2a, orange and brown lines), the O 1s features observed in the BE range of 539–542 eV are assigned to the formation of the ^3^ππ* and ^3^nπ* states (see Figure 2b), although the agreement with the theoretical spectra is not satisfactory. Nevertheless, this assignment is the most reasonable one. At very long times, we have only 3 data points, 10, 100, and 1000 ps, and some changes in the experimental spectra are evident. This may indicate slow conversion of one triplet state to the other.

The observed ISC time constant of 1.6 ± 0.4 ps falls between the two constants of 0.44 and 3.48 ps reported by Miura et al.^66^ They suggested that the presence of these two constants may result from two dynamic processes, but could also arise from the dependence of the valence cross section on the electronic and structural properties of uracil. Our calculations show that the core-level spectrum varies little during the simulation time (see Figures 2a and S14), due to motion on the S_1_(nπ*) potential energy surface. Therefore, it is more likely that the authors of ref 66 observed spectral changes resulting from variations in cross sections.

We now examine the influence of nuclear motion on the XPS signal. In the O 1s spectra in Figure 4, clear oscillations were observed, and we performed a Fourier transform analysis in order to extract their frequencies (Figure S13, Section S6, Supporting Information). In the time domain, Figure 4, oscillations in the signal intensities of the O 1s are depicted. One can see that the intensity oscillates in regions A (S_2_(ππ*)), C (S_1_/T_1_), D (S_1_(nπ*)), and E (S_1_(nπ*)), resulting in different frequency components for each domain. In particular, the main frequency modes of 114.6 cm^–1^ (291 fs), 114.8 cm^–1^ (290.5 fs), 198.1 cm^–1^ (168 fs), and 292.7 cm^–1^ (114 fs) were identified for the A, C, D, and E energy ranges, respectively (see Figure S13, Section S6, Supporting Information). From calculations, we know that the valence configuration does not change, and there is no reason to believe the Dyson norms change. We interpret these oscillations as being due to variations in the binding energy of the ionic states caused by the large anharmonic nuclear motion. For a measurement of a fixed window of kinetic energy, this results in an oscillation of the intensity as the peak maximum moves within, or partly out of, the window.

The O 1s intensity in the energy ranges A and C oscillates with a similar frequency but in antiphase and can be associated with the HGS oscillations around the ground state (range B) (see Figure S13, Section S6, Supporting Information). We will return to this point later, presenting a complementary analysis based on the scaled subtraction procedure that further supports this interpretation (see Figures S6 and S5, Supporting Information).

As for the oscillations in the ranges E and D, we correlate them to the calculated bond lengths, demonstrating satisfactory agreement with theory (Figure 5). Specifically, we analyzed the normal modes and computed the average lengths of the C5=C6 and C4=O8 bonds (Section S10, Supporting Information).

**Figure 5:**
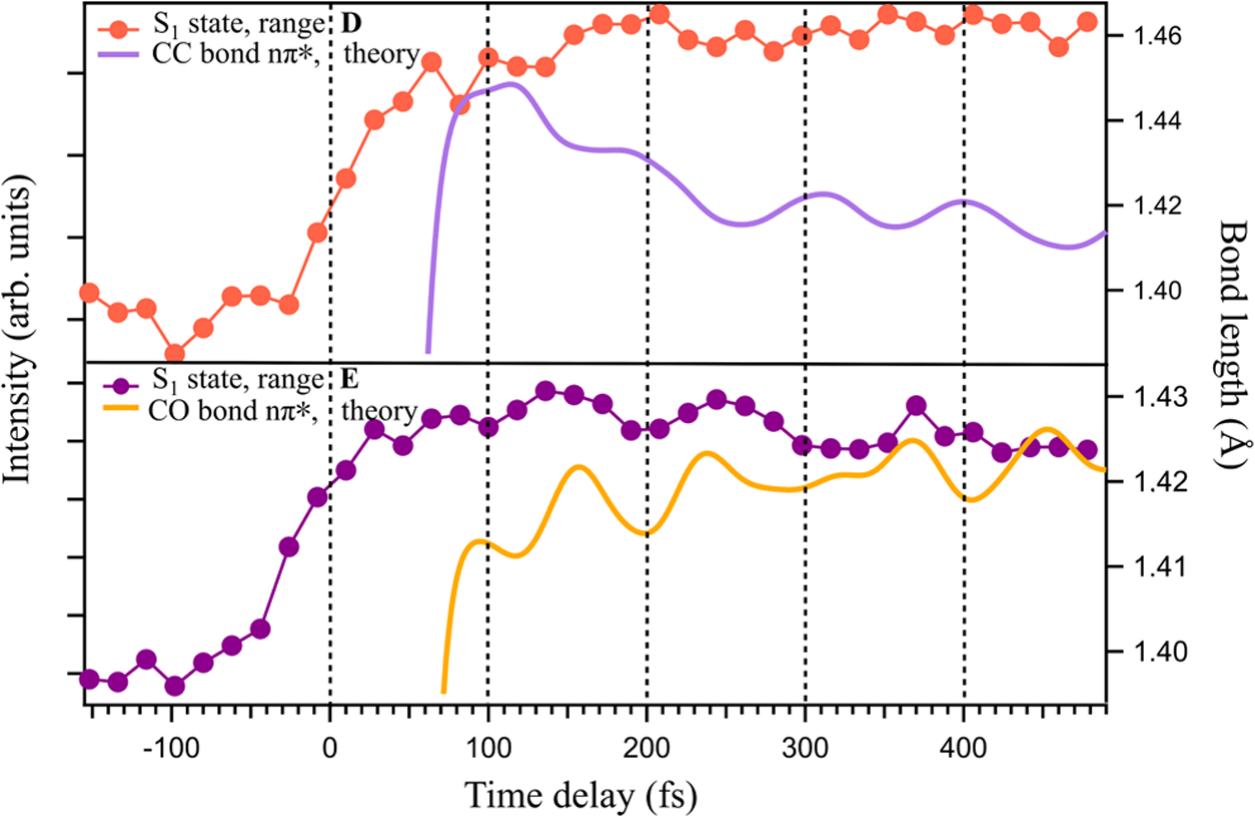
Measured O 1s intensity in energy ranges D (orange dots) and E (dark magenta dots) as a function of time delay, and calculated mean values of the C5=C6 (light purple line) and C4=O8 (yellow line) bond lengths for a set of trajectories in the S_1_(nπ*) state. Calculated curves were shifted by 30 fs in order to match the experimental data.

This approach is more accurate than comparing vibrational frequencies of the ground state, as the π → π* excitation causes a sudden ∼0.1 Å extension of the two double bonds (see Figure S17, Section S10, Supporting Information). Since the stretching of these bonds does not correspond to specific normal modes, multiple modes involved in the stretching become activated. Because the amount of internal energy and the shapes of the potential energy surfaces of the S_0_ and S_1_ states differ, the frequencies of the oscillations will be different.

At thermal energies, vibrational effects on core spectra are usually weak, but in the present case, the ground electronic state of the molecule is vibrationally excited (with an equivalent temperature of about 1800 K, assuming equipartition), and much of this energy is concentrated in the C5=C6 and C4=O8 bonds. In the π→π* transition, an electron from the conjugated π-electron system of uracil, encompassing the C5=C6 and C4=O8 bonds, is promoted to an antibonding π* orbital localized on the same moiety. The localization of the electronic excitation on the two double bonds results in a photoexcited molecule that is far from equilibrium. Consequently, the large-amplitude vibrations then modulate the core spectrum. For example, in Figure 5, the O 1s intensity for the energy ranges D and E in Figure 2b is plotted as a function of time delay together with the calculated C5=C6 and C4=O8 mean bond lengths for the ensemble of trajectories that reached the S_1_(nπ*) state (see Figure S17, Section S10, Supporting Information).

In the lower panel of Figure 5, the maxima and minima of the signal from range E correlate well with the bond length oscillations. In the upper panel, there is a hint that the signal in range D correlates with the bond length, but it is less convincing. These results indicate that the fluctuations in intensity are directly related to vibrational motion: when the C4=O8 bond length increases, the intensity in region D may increase. Simultaneously, the intensity in region E decreases, and the C5=C6 bond length also decreases. The extremely large nuclear motion affects the electronic wave functions, and this is reflected in core binding energies.

Miura et al.^66^ also observed an oscillatory signal attributed to photoionization from the nπ* state to a presumably excited valence state of the uracil ion. Their Fourier analysis indicated vibrational frequencies of 135 and 315 cm^–1^, i.e., periods of 247 and 95 fs, respectively. These periods are quite close to the values we observed, but the authors^66^ could not relate them to specific normal modes. In a very recent paper, Karashima and Suzuki^76^ associated the frequency 300 cm^–1^ (110 fs period) with the motion of the C5–H moiety, whereas we assign a similar feature to C5=C6 and C4=O8 bond stretching. The same authors reported vibrational coherence transfer during the ultrafast internal conversion from the S_2_(ππ*) to the S_1_(nπ*) state of gaseous uracil. A prominent peak at 750 cm^–1^ (44.5 fs) was found in the frequency spectra and assigned to the breathing mode of the aromatic ring, characterized by a large change in the N1C6C5 angle.^77^ This frequency is not accessible in our data due to our lower temporal resolution.

Furthermore, as noted above, we performed scaled subtraction of the O 1s spectra to generate spectra of the S_1_ state without the negative features due to the depletion of the ground state. We also removed the sideband signal at zero time delay to highlight the features associated only with the excited sample (Figure 6). The method is described in the Supporting Information (see Section S5, Supporting Information), and we found a value of *f* = 0.09, corresponding to 9% of the initial ground state population transferred to the S_1_ state. The approximations in this approach are rather crude, but the method serves to highlight some features.

**Figure 6:**
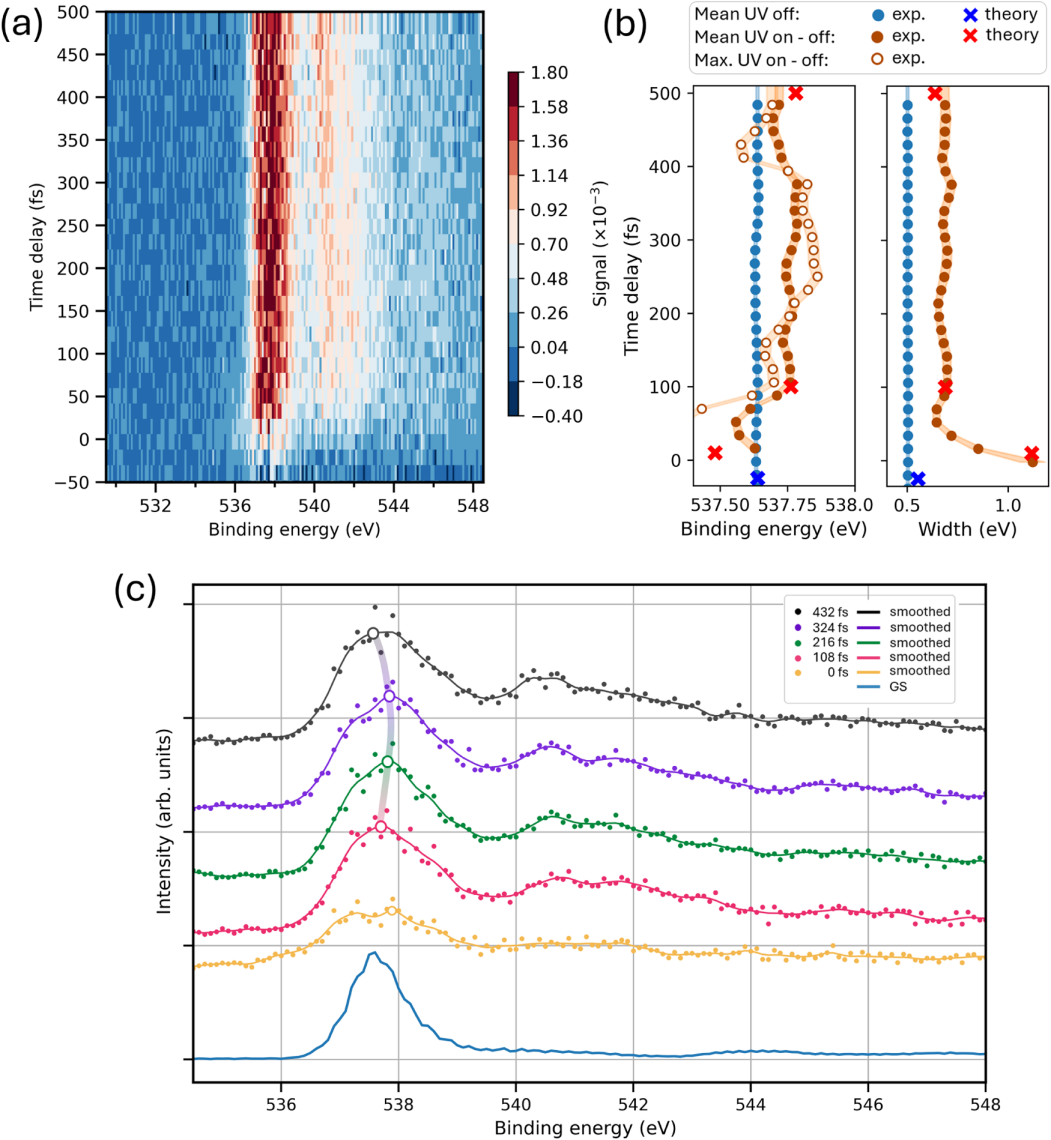
(a) False color map of O 1s after scaled subtraction, as a function of binding energy and time delay. (b) Comparison of relevant parameters of the ground state (GS) peak as a function of the pump–probe delay: UV-(on–off) (red and brown) and UV-(off) (blue and cyan). Experimental parameters are shown with circles, theoretical one with crosses. Right: the sigma width of the peak. Left: the mean (filled circles and crosses) and maximum (empty circles) value of the ground state peak. The maximum has been obtained after the Gaussian filter in the time axis with σ = 18 fs and in the energy axis with σ = 0.2 eV. (c) Bottom curve: spectrum of the GS. Upper curves: spectra of the excited state, centered at the indicated time delays and integrated over time intervals of 108 fs. The spectra have been smoothed by a Gaussian filter with σ = 0.15 eV. The maximum is indicated by an empty circle, and the gray curve connecting the maxima is shown to guide the eye.

For example, Figure 6a shows a map generated with the procedure, where the main line around 537.6 eV (core ionized ionic state) shows sensitive time-varying features in the spectral shape and position. To better highlight these features, we fitted the main line with a Gaussian and a linear baseline in the region between 536 and 539.4 eV. The mean and sigma width of the Gaussian are shown Figure 6b left and right, respectively, and they are compared with the values obtained by applying the same analysis on the experimental pump off data and on the theoretical pump–probe HGS spectrum shown in the Supporting Information (Figure S4, Section S3). A clear shift toward higher binding energy and the broadening of the GS spectrum is observed, which nicely matches with the theoretical prediction of the HGS evolution (see Figure 6b). This experimental observation alone clearly indicates that the HGS is populated after the photoexcitation. Moreover, the TR-XPS spectra generated by integrating over time intervals of 108 fs demonstrate changes in the shape as a function of time (see Figure 6c). The smoothing of these spectra reveals a small but noticeable energy drift of the maximum toward higher binding energies for 216 and 324 fs, in comparison with 432 and 108 fs (empty circles). When applying the same procedure with a finer temporal resolution (Figure 6b, empty circles), we observe an oscillatory behavior of the maximum that correlates with the previously reconstructed oscillations in two energy regions (A and C) close to the GS. Figure 6b,c shows that at around 150 and 420 fs, the maximum moves toward lower binding energy. Taking into account that the spectral width of the HGS state is ≈1.6 eV (fwhm), this can explain the increased contribution in the low-energy tail of the HGS (region A) around the same pump–probe delays. Similarly, at approximately 250 fs, the peak moves toward higher binding energy, which may explain the concomitant increased contribution observed in the high-energy tail of the HGS (region C). Hence, our experimental data confirm the prediction of our calculations that the core spectra are not constant in time, but oscillate during the relaxation process.

## Conclusions

In this study, time-resolved X-ray photoelectron spectroscopy (TR-XPS) was employed to directly probe the oxygen and nitrogen atoms in uracil, uncovering its relaxation dynamics following UV excitation. While all core levels contributed to understanding the dynamics, the O 1s signal emerged as particularly informative. Our findings demonstrate that TR-XPS spectra capture both changes in charge distributions and structural changes in the excited states. The present experimental data are in good agreement with computed XPS spectra for the ground and excited states, performed on structures sampled from surface hopping nonadiabatic dynamics simulations.

The photoinduced dynamics of uracil take place on three time scales: (i) an ultrafast step of tens of fs, (ii) an intermediate step of around 1.6 ps, and (iii) a slow relaxation, greater than 10 ps. In the first interval, two ultrafast processes occur in parallel, one being the formation of vibrationally excited ground state molecules, which occurs via the direct S_2_(ππ*) → S_1_(ππ*) → S_0_ deactivation pathway. As well, the indirect deactivation channel S_2_(ππ*) → S_1_(nπ*) occurs via internal conversion with a time constant 17 ± 4 fs. In the second interval, a single process occurs: S_1_(nπ*) → *T*_1_/*T*_2_ with a time constant of 1.6 ± 0.4 ps. Lastly, on the time scale of 10–1000 ps, we observe spectral changes, which we tentatively assign to conversion between triplet states. This confirms that the nπ* state is the doorway for ISC relaxation to the triplet states, responsible for the slower picosecond/nanosecond dynamics of uracil.

The results provided by the present work clearly demonstrate that TR-XPS spectra are also capable of following nuclear dynamics effects. The observed oscillations in the signal intensity of the O 1s (at fixed kinetic energy) are directly related to the vibrational motion of the C5=C6 and C4=O8 bond lengths during the ultrafast electronic relaxation processes of uracil.

## Supplementary Material



## Data Availability

The raw data recorded for the experiment at the European XFEL are available at: 10.22003/XFEL.EU-DATA-003014-00.

## Notes

Experimental and theoretical carbon K-edge TR-XPS spectra of uracil (see Section S12); details concerning experimental data processing and time-resolved fitting; and additional figures, tables, and structures that support conclusions presented in the article (PDF)
