# Supplementary material for: Unraveling the Relaxation Dynamics of Uracil: Insights from Time-Resolved X‑ray Photoelectron Spectroscopy
Source: J Am Chem Soc. 2025 Aug 13;147(34):30694–707. doi: 10.1021/jacs.5c04874 (PMC12395411; doi:10.1021/jacs.5c04874)
Supplement: Supplementary file 1 [file ja5c04874_si_001.pdf]

Supporting Information:

Unraveling the relaxation dynamics of Uracil: insights from time-resolved X-ray photoelectron spectroscopy

Davide Faccialà,[†,‡] Matteo Bonanomi,[¶,‡] Bruno Nunes Cabral Tenorio,[§,||] Lorenzo Avaldi,[⊥] Paola Bolognesi,[⊥] Carlo Callegari,[#] Marcello Coreno,[⊥] Sonia Coriani,[*,§] Piero Decleva,[@] Michele Devetta,[△] Nađa Došlić,[*,∇] Alberto De Fanis,[††] Michele Di Fraia,[‡‡,#] Fabiano Lever,[¶¶,§§] Tommaso Mazza,[††] Michael Meyer,[††] Terry Mullins,[††] Yevheniy Ovcharenko,[††] Nitish Pal,[#] Maria Novella Piancastelli,[||||,⊥⊥] Robert Richter,[#] Daniel E. Rivas,[††] Marin Sapunar,[∇] Björn Senfftleben,[††] Sergey Usenko,[††] Caterina Vozzi,[△] Markus Gühr,[¶¶,§§,##] Kevin C. Prince,[*,#] and Oksana Plekan[*,#]

†*CNR-IFN, Istituto di Fotonica e Nanotecnologie, 20133 Milano, Italy*

‡*these authors contributed equally*

¶*Dipartimento di Fisica, Politecnico di Milano, 20133 Milano, Italy*

§*Department of Chemistry, Technical University of Denmark, DK-2800 Kongens Lyngby, Denmark*

||*Departamento de Química, Universidad Autónoma de Madrid, Madrid 28049, Spain*

⊥*Istituto di Struttura della Materia-CNR, 00133 Rome, Italy*

#*Elettra-Sincrotrone Trieste S.C.p.A., in Area Science Park, 34149 Basovizza, Trieste, Italy*

@*Dipartimento di Scienze Chimiche e Farmaceutiche, Universitá degli Studi di Trieste, I-34127 Trieste, Italy*

△ *CNR-IFN, Istituto di Fotonica e Nanotecnologie, 20133 Milano, Italy*

∇*Institut Ruđer Bošković, Bijenička cesta 54, 10000 Zagreb, Croatia*

††*European XFEL, Holzkoppel 4, 22869 Schenefeld, Germany*

‡‡*CNR - Istituto Officina dei Materiali (IOM), in Area Science Park, 34149 Basovizza, Trieste, Italy*

¶¶*Institut für Physik und Astronomie, Universität Potsdam, 14476 Potsdam, Germany*

§§*Deutsches Elektronen-Synchrotron DESY, Notkestraße 85, D-22607 Hamburg, Germany*

||||*Sorbonne Université, CNRS,Laboratoire de Chimie Physique-Matiere et Rayonnement, LCPMR, Paris F-75005, France*

⊥⊥*Department of Physics and Astronomy, Uppsala University, Uppsala SE-75120, Sweden*

##*Institut für Physikalische Chemie, Fachbereich Chemie, Universität Hamburg, 20146 Hamburg, Germany*

E-mail: soco@dtu.dk; nadja.doslic@irb.hr; kevin.prince@elettra.eu; oksana.plekan@elettra.eu

Contents

S1 Nonadiabatic dynamics simulations and calculation of the time-resolved O 1s and N 1s spectra of uracil. S-3

S2 Calculated O 1s spectra at the ground state equilibrium geometry. S-8

S3 Spectrum of the hot ground state (HGS). S-10

S4 Supporting figures for the experimental section. S-11

S5 Procedure for scaled subtraction. S-16

S6 Procedure used for fitting the O 1s TR-XPS spectra of uracil. S-18

S7 Direct relaxation pathway of uracil. S-30

S8 Calculated O 1s difference spectra. S-31

S9 Depletion of the N 1s signal. S-32

S10 Calculated average bond lengths. S-33

S11 Geometries of the relevant minima and conical intersections. S-35

S12 Time-resolved C 1s spectra of uracil. S-36

S1 Nonadiabatic dynamics simulations and calculation of the time-resolved O 1s and N 1s spectra of uracil.

Nonadiabatic dynamics simulations were carried out using the fewest switches surface hopping method.[S1] Calculations, restricted to singlet electronic states, were performed by Milovanović et al.[S2] using the spin-component scaling (SCS) variant[S3,S4] of the second-order algebraic-diagrammatic-construction (ADC(2)) method[S5,S6] and the aug-cc-pVDZ basis set with an in-house code[S7] linked to the Turbomole program package.[S8]. ADC(2) is a single-reference method based on the MP2 description of the electronic ground state and is therefore considered an excited-state extension of MP2. It accurately captures the topography of CoIns between excited states, making it well-suited for nonadiabatic simulations of small to medium-sized systems. However, since ADC(2) excited states are not coupled to the MP2 ground state, the method becomes unreliable near CoIns involving the ground and first excited states. In this work, we employ the SCS-ADC(2) variant to mitigate the influence of Rydberg states, whose excitation energies are typically underestimated by standard ADC(2),[S9] on the dynamics.

To allow the possibility of crossing to the triplet manifold, calculations were repeated with the SHARC 3.0 program.[S10,S11] The required spin-orbit matrix elements were computed using the spin-orbit mean field (SOMF) method implemented in the Orca 5.0 program package.[S12] Initial geometries and momenta were selected from 1000 randomly sampled geometries according to their oscillator strengths in the 4.7-4.8 eV excitation window. In all cases, the calculations were initiated in the $S_2(\pi\pi^*)$ state. Newton's equations for nuclear motion were integrated for 1000 fs with time steps of 0.5 fs, using the velocity-Verlet algorithm. The local diabatization formalism was used to propagate the electronic wave function and compute the hopping probabilities.[S13] The energy-based decoherence procedure of Granucci and Persico[S14] with $\alpha = 0.1\ E_h$ was used.

Time-resolved O 1s and N 1s XPS spectra of uracil were calculated using an ensemble

of 48 SH (surface-hopping) trajectories.[S2] Table S1 provides an overview of the distribution of SH trajectories between the $\pi\pi^*$ and $n\pi^*$ states at specific propagation times, illustrating the dynamic evolution of the ensemble.

Table S1: Distribution of an ensemble of 48 SH trajectories in the two valence excited states of uracil ($\pi\pi^*$ and $n\pi^*$) and the electronic ground state S_0 at specific delay times. The ensemble was used to compute the O 1s and N 1s spectra shown in Figure 2(a) and Figure 3(a), respectively (main text). The subset of trajectories that deactivate to S_0 contributes to the hot ground state signal (HGS) (see below, Fig. S4).

Time (fs)	$\pi\pi^*$	$n\pi^*$	S_0
0	48	0	0
10	48	0	0
25	43	5	0
50	40	7	1
150	15	23	10
400	0	31	17

The partial atomic charges in the S_2 and S_1 excited states of uracil computed with RASPT2 from Mulliken population analysis are presented in Table S2. The transition to the $S_2(\pi\pi^*)$ state, which is conveniently described in terms of natural transition orbitals (NTOs) shown in Fig. S1, is accompanied by a flow of valence electrons. Specifically, electrons migrate from the bonding π orbital primarily localized on the O8, O7, N1, and C5=C6 groups towards the antibonding π^* orbital of the carbonyl group. Consequently, this electron redistribution leads to a migration of electron density from O7 and N1 towards the region encompassing N3 and the carbonyl group C4=O8. In contrast, the $S_2 \rightarrow S_1$ internal conversion leads to a significant reduction of the electron density on the O8 atom and a slight increase of electron density on the nitrogen atoms. On these grounds, the excess or deficit of valence electron density on oxygen or nitrogen is anticipated to result in a red-shift or blue-shift, respectively, of the corresponding core binding energies in the XPS spectra.[S15]

Indeed, the experimentally observed intensity around 536 eV (see Figure 2(b), range A, main text) assigned to the core ionization of O8 in the S_2 bright state of uracil was found to be red-shifted with respect to the GS peak, due to the effect of an increase of electron

Table S2: Partial charges from RASPT2 calculations at the FC geometry using the aug-cc-pVDZ basis set. RASPT2 partial charges are given by the difference between the Mulliken charge of the corresponding atom in the excited state minus the Mulliken charge of the atom in the ground state (GS).

	O7	O8	N3	N1	C6	C2	C4	C5
δ_{S_0}	−0.72	−0.81	−0.48	−0.53	0.77	1.08	0.72	0.80
δ_{S_1}	−0.66	−0.47	−0.54	−0.55	0.68	1.10	0.63	0.72
δ_{S_2}	−0.66	−0.76	−0.53	−0.46	0.73	1.06	0.67	0.79
$\delta_{S_1-S_0}$	0.06	0.34	−0.06	−0.02	−0.09	0.02	−0.09	−0.08
$\delta_{S_2-S_0}$	0.06	0.05	−0.05	0.07	−0.04	−0.02	−0.05	−0.01

density on the carbonyl group C4=O8 (see Figure 1(b) and Table S2). Conversely, the broad feature caused by the deficit of electron density on O8 in the $S_1(n\pi^*)$ state displays a notable blue-shift, as illustrated in the 2D map (see Figure 2(b), range C-E, main text). These experimental observations are in good agreement with our computations.

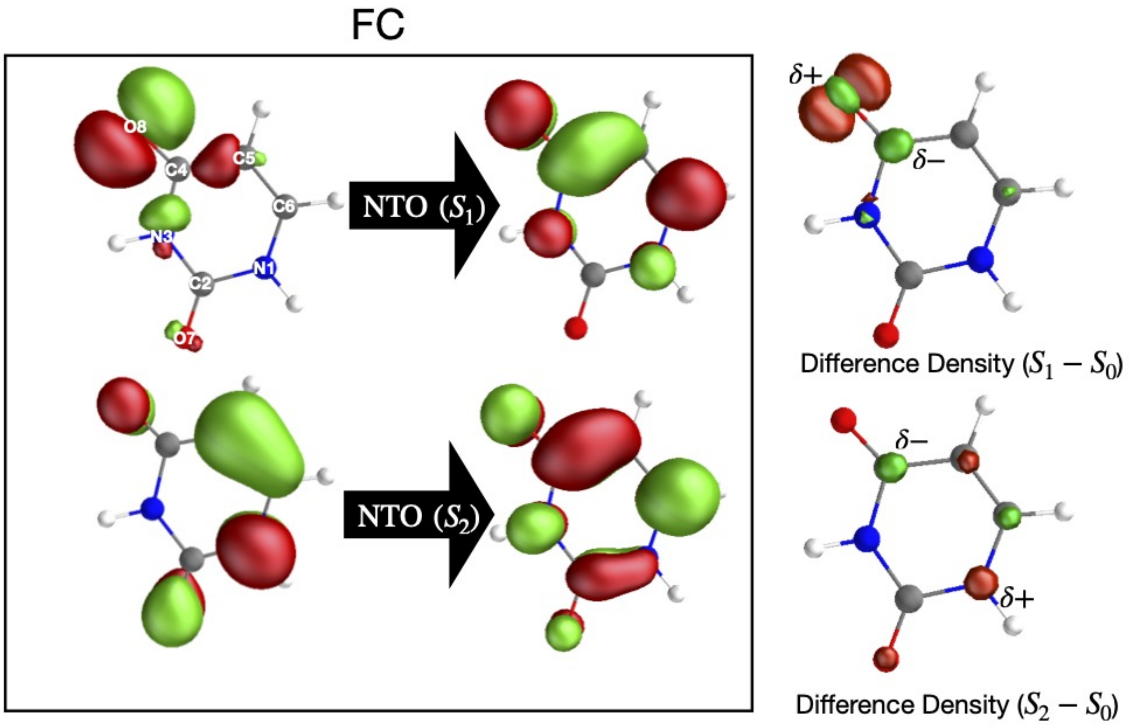

Figure S1: Natural transition orbitals (NTOs) of the valence excited states $S_1(n\pi^*)$ and $S_2(\pi\pi^*)$ computed at the ground state equilibrium geometry (left), and density differences (right) between each valence excited state and the ground state. The signs of partial charges "$\delta + /-$" are indicated, and numerical values are given in Table S2.

As for the N 1s spectra, the computed partial atomic charges at the FC geometry show a significant decrease of the electron density at the N1 atom in the $S_2(\pi\pi^*)$ state and an increase at the N3 atom (see Table S2). Hence, a blue-shift relative to the GS is expected for the optically bright state in the N1s TR-XPS spectra. However, the signature of the bright state was only predicted theoretically, but not observed experimentally due to the lack of instrumental resolution (see Figure 3(a), blue dashed line, main text). In accord with our calculations, the partially positive charge on the N1 atom in the $S_2(\pi\pi^*)$ excited state in the FC region disappears for distorted geometries at 50 fs (gray), that is, for geometries at which the $S_2(\pi\pi^*)/S_1(n\pi^*)$ internal conversion takes place (see Figure 3(a), main text). The simulated spectra for time delays of 150 and 400 fs, where signal is exclusively due to the dark state, demonstrate an additional shift towards lower binding energy compared to the ground state spectra. Indeed, the experimentally observed signature of the S_1 state of uracil was found to be red-shifted with respect to the GS, which is in line with the increase of electronic density on the N3 atom for this state (see Figure 3(b), range A, main text).

In addition, Fig. S2 shows the active subspaces utilized in the restricted active space self-consistent field (RASSCF) calculations (see main text). RAS1 comprises the relevant core orbitals, RAS2 includes seven valence-occupied orbitals, and RAS3 is formed by two π^* orbitals, each capable of accommodating a maximum of two electrons.

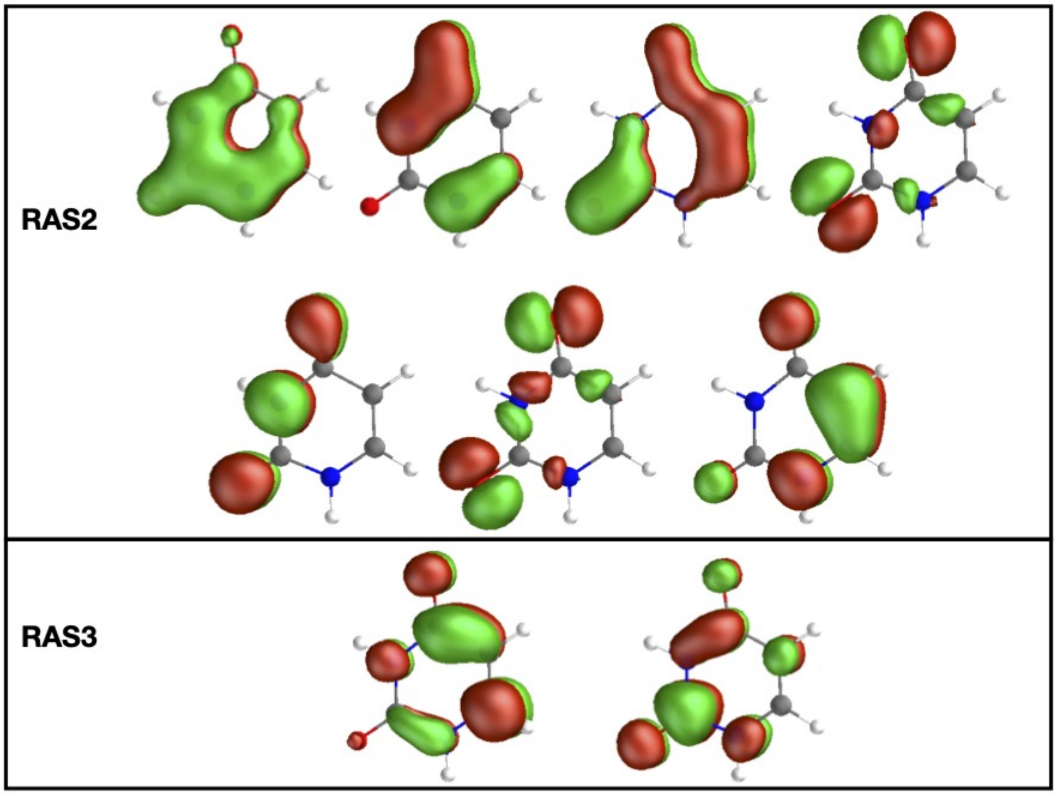

Figure S2: Active space used in the RASSCF calculations in a three-fold division of the active space into RAS1, RAS2, and RAS3. The RAS1 subspace (not shown) consists of the pertinent core orbitals for which a single-hole configuration is enforced. The RAS2 subspace contains seven valence-occupied orbitals. Finally, the RAS3 subspace is formed by two π^* orbitals and accepts a maximum of two electrons. The orbitals shown are state-averaged over ten neutral states.

S2 Calculated O 1s spectra at the ground state equilibrium geometry.

O 1s XPS spectra calculated at the equilibrium geometry are shown in Fig. S3. The cross sections have been obtained with an explicit description of the electronic continuum with an LCAO B-spline basis using the Tiresia code.[S16] Electronic exchange and correlation effects in the continuum have been accounted for by the LB94[S17] functional. The continuum states were described by a large one-center expansion of B-splines enclosed in a sphere of 25 a.u. with origin at the center of mass, using spherical harmonics of angular momentum up to $l_{max} = 20$ to get well converged results. A small off-center expansion located over the nuclei varied from 0.5 to 1.0 a.u., larger for the heavier nuclei, and an angular expansion limited to $l_{max} = 2$.

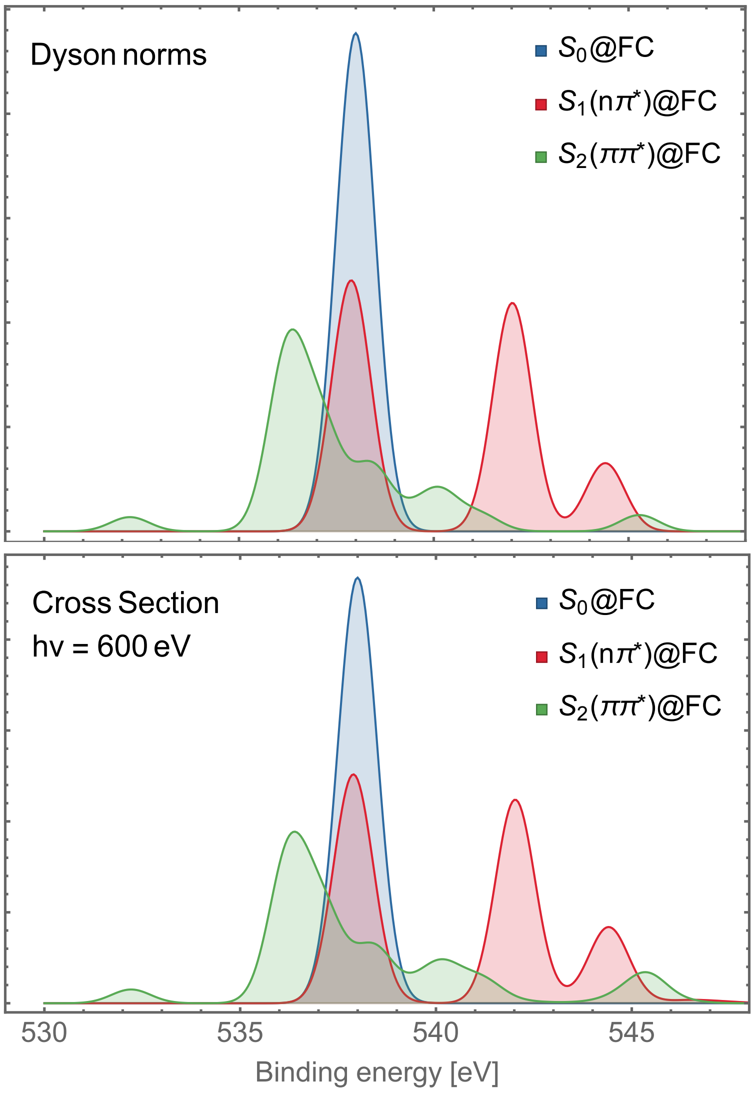

Figure S3: Calculated O 1s XPS spectra using the equilibrium geometry of the ground state.Top panel: convolution of the computed ionization energies and Dyson norms with a Lorentzian function (FWHM = 0.4 eV). Bottom panel: spectrum obtained by convolution of the computed ionization energies and the cross sections computed for a photon energy of 600 eV.

S3 Spectrum of the hot ground state (HGS).

To compute the ground state bleach signal we have performed MP2-based dynamics simulations in the electronic ground state using the same initial conditions as in SH simulations ($t = 0$). The simulations provide the time evolution of the reference ground-state thermal ensemble. In addition, a second set of simulations was performed for the subset of trajectories that during SH simulations ended in the ground state giving rise to the HGS signal see Fig. S4. To simulate the time evolution of this HGS ensemble, initial conditions were obtained from the final $S_1(\pi\pi^*)/S_0$ CoIn geometries and velocities in the SH simulations.

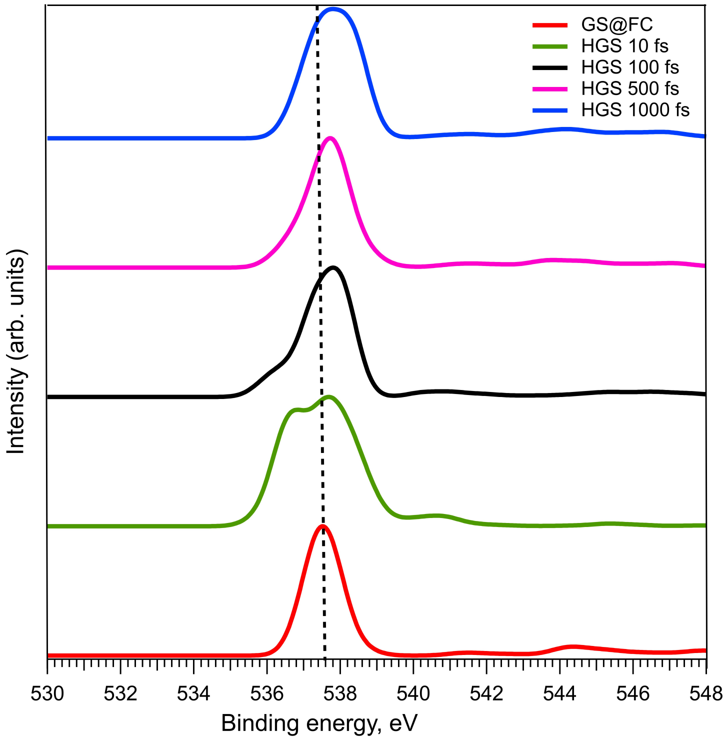

Figure S4: Calculated O 1s XPS spectra for a set of geometries sampled from a ground state trajectory (not excited by the UV laser, red) and from nonadiabatic trajectories that relaxed back to the ground state (colors). The trajectories are synchronized in such a way as to start from the $S_1(\pi\pi^*)/S_0$ CoIn at $t = 0$. The O 1s XPS spectra are computed at 10 fs (green), 100 fs (black), 500 fs (purple) and 1 ps (blue). All spectra are shifted by 2.4 eV to lower binding energy. The vertical black dashed line indicates the energy of the experimental GS spectrum at 537.6 eV.[S18]

S4 Supporting figures for the experimental section.

The experiment was conducted at the Small Quantum Systems (SQS) instrument located at the SASE3 undulator of the European XFEL[S19]. The XFEL beam consisted of 10 trains of pulses per second, with 166 pulses per train at a repetition rate of 376 kHz within the train. The undulator was tuned to provide X-ray pulses at 600 eV with a 5 eV full width at half maximum (FWHM) bandwidth and mean pulse energy of 6.8 mJ. The FEL pulses passed through a gas attenuator reducing their energy to 238 μJ[S20]. The FEL beam had an estimated duration of approximately 30 fs as inferred from the electron bunch charge of 250 pC[S21,S22] and the focus diameter of 100 μm (FWHM).

The SQS instrument monochromator[S23] was used to reduce the FEL bandwidth to 0.136 eV (FWHM), which corresponds to a resolving power of $E/\Delta E = 4.4\times10^3$ and resulting in a pulse energy of 0.21 μJ on the target (see Fig. S5). The same monochromator is also utilized as a spectrometer for spectral diagnostics of the FEL beam. The spectrometer operation mode is realized by introducing a YAG:Ce crystal into the focal plane of the monochromator[S24]. Figure S6 shows the well-known atomic Ne 1s–3p X-ray absorption resonance at 867.12 eV, used to calibrate the monochromator energy scale[S25].

Moreover, in the experimental ground state O 1s spectrum,[S18] the two non-equivalent oxygen core levels were not resolved, and appeared as a single peak with a maximum at 537.6 eV. We applied a fine calibration (0.1 - 0.2 eV) to the energy scale using the literature value to account for possible drifts of the monochromator or spectrometer. The offset was determined from ground state spectra and applied to them and to the following excited state spectra.

Uracil was excited to its lowest-energy $\pi\pi^*$ absorption band[S2] by the third harmonic of an optical laser operating at $\approx$ 800 nm[S26] and synchronized to the X-ray pulses. The central wavelength of the pump pulses was 264 nm and the focus diameter was 150 μm (FWHM). The duration of the UV pulses (approximately 75 fs) was measured by cross-correlation

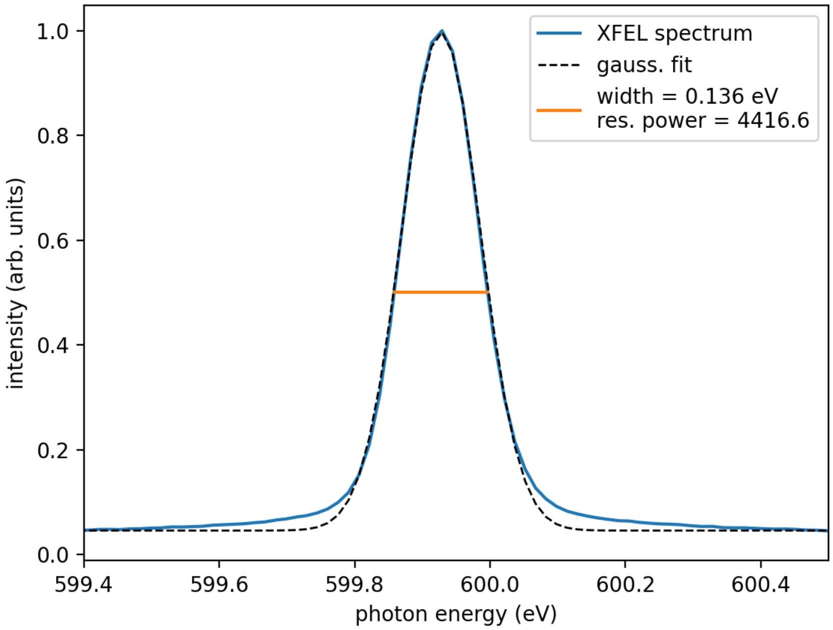

Figure S5: FEL spectrum as the output of the SQS instrument monochromator measured at the spectrometer operation mode at 600 eV. Average of 236 shots. A fit with a Gaussian function is shown.

measurement[S27] (see Fig. S7).

The initial temporal and spatial overlap between UV and soft X-ray lasers were optimized using a higher pump energy and observing the depletion of the normal Auger signal of uracil. The depletion was induced by fragmentation at full UV energy (11 μJ per pulse) which leads to photoproducts with different Auger spectra. Subsequently, a more precise temporal overlap condition was found and routinely checked by monitoring the formation of laser-induced sidebands (SBs) around the 1s photoline. The presence of sideband signal occurring in TR-XPS spectra around t=0 delay was always ascertained in order to ensure the temporal overlap between both UV and X-ray pulses. In addition, the SB signal yielded the precise pump–probe instrument response function resulting in a value of 80.5 fs for the cross-correlation of the temporal widths of the pump and probe (see Fig. S7)).

During the experiment, photoion time-of-flight (TOF) mass spectra were measured with different laser intensities in order to check that the molecules were not excessively pumped

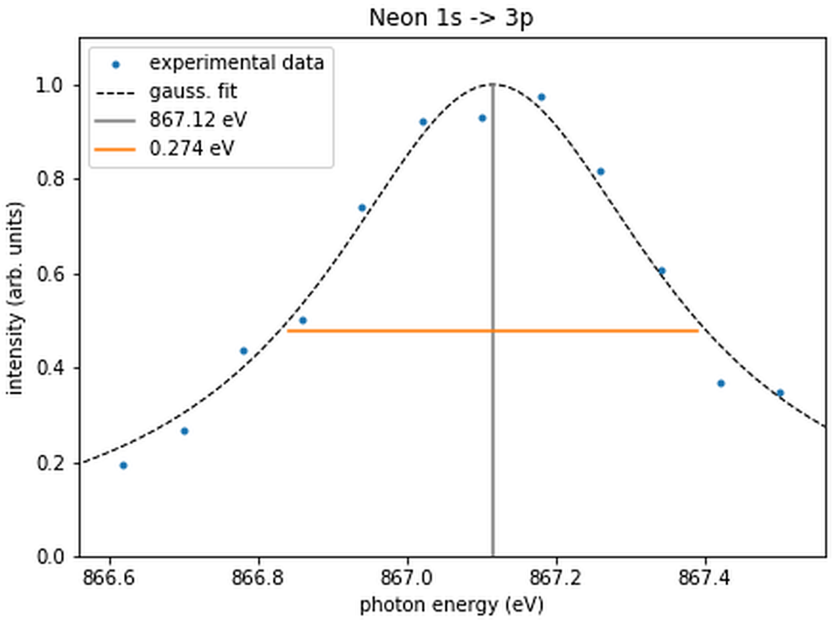

Figure S6: Ne 1s–3p X-ray absorption spectrum used to calibrate the monochromator. An offset of 0.44 eV with respect to the nominal energy was found.

by the UV laser. The parent ion showed a quadratic dependence on the laser intensity, as expected for weak pumps. Some of the fragments (e.g., mass 69) showed saturation at UV pulse energy $> 8\mu$J. Hence, in the present experiment the UV pump energy was set to 5μJ in order to avoid the fragment saturation region (see Fig. S8).

Uracil was purchased from Sigma Aldrich and evaporated without further processing from an effusive capillary oven[S28] at a temperature of 160 °C into an ultra-high vacuum chamber, creating a molecular beam that interacts with two photon beams (optical and X-ray) in the center of a magnetic bottle electron spectrometer (MBES). This spectrometer provides highly efficient time-of-flight measurements of electrons emitted into 4π solid angle, with nearly 100 % angular acceptance. Its operating principle is similar to other units described elsewhere.[S29–S31] The measured time-of-flight spectra were converted to an energy scale and binned at intervals of 100 meV.

The MBES spectrometer was calibrated by measuring Ne 1s spectra while varying the photon energy. In particular, we were scanning the photon energy so that the Ne 1s pho-

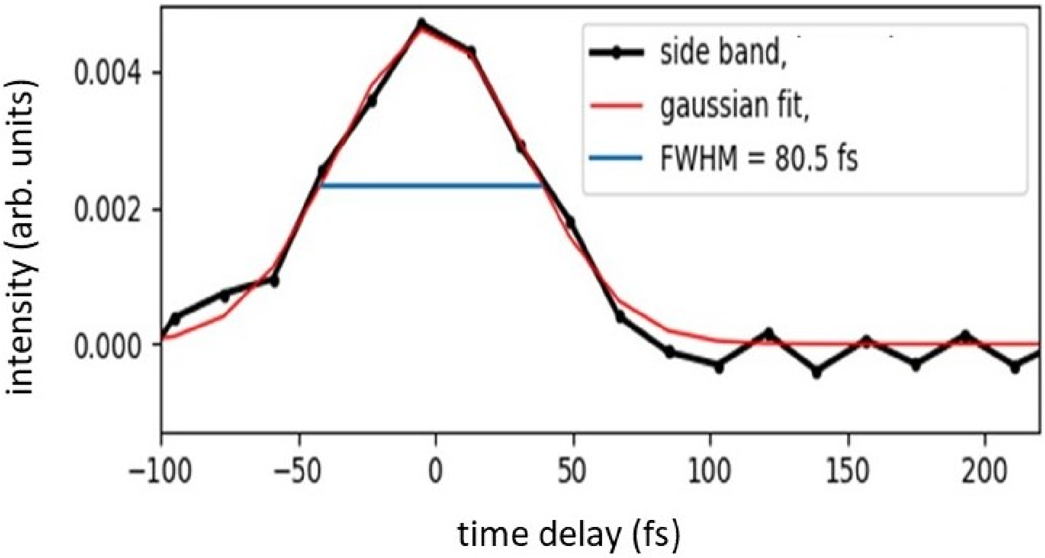

Figure S7: The cross-correlation function between the UV optical and FEL laser pulses.

toline[S25] fell in the same kinetic energy ranges as those used for the O 1s, N 1s and C 1s photolines at 600 eV photon energy. Three different retardation voltages of −45 V, −180 V and −302 V were applied to the electrostatic lens of the MBES in order to obtain high resolution photoelectron spectra at the oxygen, nitrogen and carbon K-edges, respectively.

The resolution of the MBES was estimated from the photoionization of Ne 1s (binding energy 870.2 eV), by scanning the photon energy of the XFEL in the energy region between 911 eV and 1048 eV. A retardation of 45 V was applied. Figure S9 shows the measured relative resolution $\Delta E/E$ (ΔE is the FWHM and E is the kinetic energy as measured, i.e., not compensated for the retardation potential) as a function of E which was fitted with the equation:

$$F(x) = A + B/x^C \tag{S1}$$

where x is the kinetic energy and F is the relative resolution ($\Delta E/E$). The asymptotic relative resolution for kinetic energies > 20 eV was estimated to be $\Delta E/E \approx 0.03$.

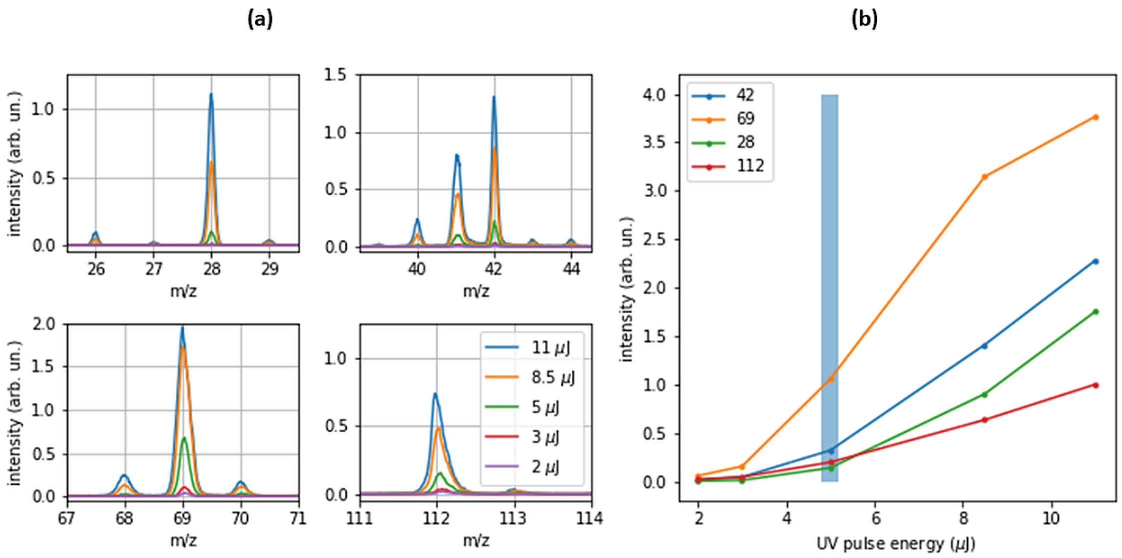

Figure S8: (a) Measured TOF spectra of uracil as a function of UV pulse energies (only main fragments are shown). (b) UV dependence of signal for the selected fragments. The blue bar indicates the operating optical laser energy.

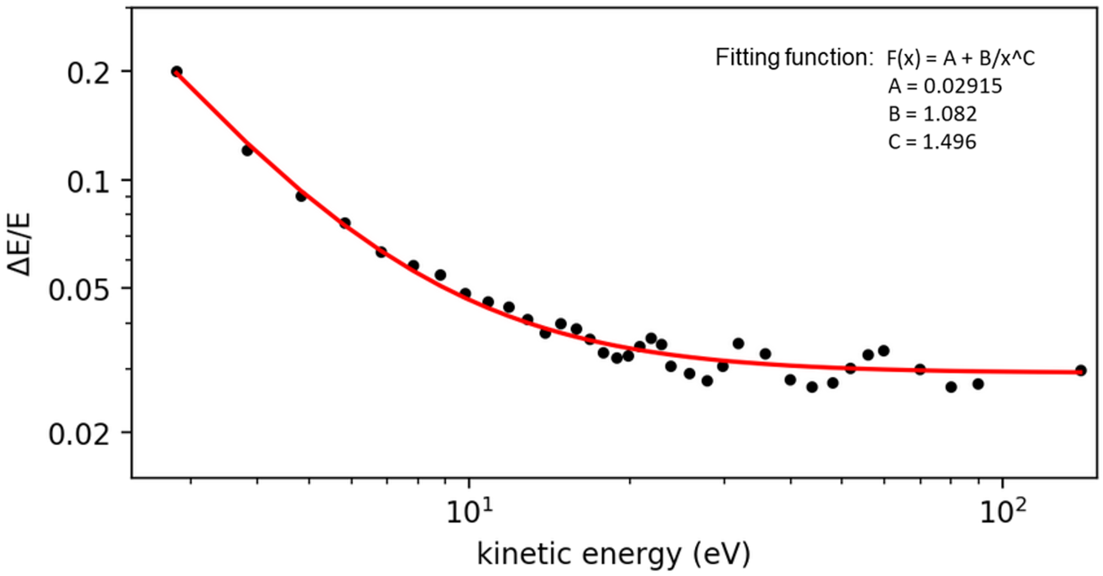

Figure S9: Measured relative resolution ($\Delta E/E$) as a function of kinetic energy E after the retardation.

S5 Procedure for scaled subtraction.

We analysed the O 1s spectra using scaled subtraction of the ground state spectrum, to generate the spectra of the S_1 state without the features due to depletion of the ground state. The latter appear as negative structures when the ground state spectrum is subtracted from the excited state spectra without taking account of depletion. The raw data also contained an O 1s peak due to residual water, which subtracts out when the difference is taken without scaling. For scaled subtraction, a residue of this peak may remain in the spectrum as a negative feature, so the data were corrected by fitting the water peak and subtracting it. We know from calculation that the O8 1s binding energy increases strongly in the S_1 state, while the O7 1s binding energy changes very little. We integrated the intensity of the ground state spectrum over the main line (O7 and O8) in the range 536 to 539.6 eV and over the shake-up range, from 539.6 to 549 eV, and found the ratio of intensities was 0.71:0.29. In the S_1 state, the theoretical cross sections on Figure S3 (red curve) predict that the main line is reduced by 48%, while 65.5% of the intensity in the ground state main line is transferred to the shake-up region, with the total not summing up to 100% due to the higher total intensity in the excited state. Taking into account that the SH calculations predict that 36% of the excited sample quickly returns to the HGS, and assuming that the shake-up intensity is unchanged in the S_1 state, a ratio of 0.46:0.54 is expected for the main line (O7) to shake-up plus O8 intensities. The ground state spectrum was then scaled and subtracted from the excited state spectra; the scaling factor was chosen to give the expected ratio, and had a value of $f = 0.09$. As an example, Figure 6(a) (see main text) shows a map generated with this procedure. In this map, we also removed the time-zero features attributed to the sideband (SB). This was achieved by fitting the isolated sideband at 532.9 eV with a 2D Gaussian. The resulting fit was then used to subtract both sidebands observed at 532.9 eV and 542.3 eV, as well as the negative depletion of the main peak at time zero due to the sideband process, assuming that this depletion corresponds to twice the amplitude of each

individual sideband.

The integral of the O 1s core level (as well as the N 1s) signal of the photoexcited sample, including the depleted ground state signal, was compared to the integral of the ground state spectrum and was found to be equal within experimental error. This rules out an excessive pump pulse energy which may have led to 2-photon ionization, producing artifacts in the experimental spectrum. Core ionization of valence ionized molecules produces photoelectrons whose energy is shifted outside the measured energy window (ionized molecules have higher ionization potential than the corresponding neutral molecule), so that, in the presence of strong valence ionization by the pump laser, a decrease in the overall signal of the photoexcited sample is expected. Since the difference of the integrated signals of the ground and excited states was zero, we conclude that the fraction of the sample ionized by the pump laser only is negligible compared with the neutral photoexcited fraction.

S6 Procedure used for fitting the O 1s TR-XPS spectra of uracil.

We modeled the observed dynamics by a set of ordinary differential equations that describe the evolution of populations in each state over time. We indicate with $g(t, t_0, \sigma)$ the effective temporal envelope of the UV laser pulse triggering the observed dynamics, where t_0 is the pump-probe time zero and σ the standard deviation of the pulse. This term incorporates the cross-correlation of the pump and probe pulses, capturing both the excitation profile and the experimental temporal resolution. It is assumed to be a Gaussian:

$$g(t, t_0, \sigma) = \frac{\exp\left(-\frac{(t-t_0)^2}{2\sigma^2}\right)}{\sigma\sqrt{2\pi}} \tag{S2}$$

The population dynamics of the ground state $S_0(t)$, the initially excited state $S_2(t)$, the lowest-energy excited state $S_1(t)$, and the triplet state $T(t)$ are described by the following set of differential equations:

$$\begin{aligned} \frac{dS_0(t)}{dt} &= -g(t, t_0, \sigma) \\ \frac{dS_2(t)}{dt} &= g(t, t_0, \sigma) - \frac{S_2(t)}{t_1} \\ \frac{dS_1(t)}{dt} &= \frac{S_2(t)}{t_1} - \frac{S_1(t)}{t_2} \\ \frac{dT(t)}{dt} &= \frac{S_1(t)}{t_2} \end{aligned} \tag{S3}$$

where t_1 is the decay time from the singlet S_2 to S_1 state, and t_2 is the decay time from the S_1 to the triplet T state. Eq. (S3) was solved numerically using the `solve_ivp` function from the `scipy.integrate` library. The free parameters t_1, t_2, σ, and t_0 were optimized through a global fitting procedure to reproduce the time-dependent dynamics observed in the energy ranges presented in Figure 2(b) up to 500 fs (main text).

Figure S10 shows the result of the fit for the six energy regions, while Table S3 summarizes best-fit values of all parameters. For the global fit, $N_j = 6$ time-dependent functions $f_j(t)$ were considered, with $j = \mathrm{SB}$ (sideband), $\mathrm{A, B, C, D, E}$ indicating the energy ranges over which the signal was integrated (see Figure 2(b) of the main manuscript). Specifically, $f_{\mathrm{SB}}(t)$ was obtained by integrating in the range $531.9 - 534$ eV, $f_{\mathrm{A}}(t)$ in the range $535.2 - 536.6$ eV, $f_{\mathrm{B}}(t)$ in the range $537.2 - 538$ eV, $f_{\mathrm{C}}(t)$ in the range $539 - 540$ eV, $f_{\mathrm{D}}(t)$ in the range $540.1 - 541.5$ eV, and $f_{\mathrm{E}}(t)$ in the range $541.6 - 543.5$ eV.

Table S3: Fitted parameters and their errors. The amplitudes and amplitude errors are shown in arbitrary units, but they have all been scaled by the same common factor to show the relative weight that each state has in a certain energy region.

**Parameter**	**Fitted Value**	**Error**
t_0 (fs)	81.14	1.7
σ (fs)	34.26	1.4
t_1 (fs)	17.03	3.7
t_2 (fs)	1585.40	377.5
$a_{SB,g}$	0.47	0.022
a_{A,S_2}	0.88	0.18
$a_{A,erf}$	0.021	0.005
a_{B,S_0}	1.15	0.023
$a_{B,g}$	0.36	0.058
a_{C,S_1}	0.24	0.023
$a_{C,T}$	0.53	0.14
a_{D,S_1}	0.76	0.034
$a_{D,T}$	0.93	0.20
a_{E,S_1}	1.12	0.045
$a_{E,g}$	0.35	0.045

Guided by the calculated spectra shown in Figure 2(a) (main text), only specific states were considered for each band, and the contribution (amplitude) of each state was fitted accordingly. The amplitude contribution of the i-th state to the j-th energy range is denoted as $a_{j,i}$. The following equations describe the time-dependent contributions for each energy

range:

$$
\begin{aligned}
f_{\mathrm{SB}}(t) &= a_{\mathrm{SB},g} \cdot g(t) \\
f_{\mathrm{A}}(t) &= a_{\mathrm{A},S_2} \cdot S_2(t) + a_{\mathrm{A,erf}} \cdot \mathrm{erf}(t) \\
f_{\mathrm{B}}(t) &= -a_{\mathrm{B},g} g(t) + a_{\mathrm{B},S_0} S_0(t) \\
f_{\mathrm{C}}(t) &= a_{\mathrm{C},S_1} S_1(t) + a_{\mathrm{C},T} T(t) \\
f_{\mathrm{D}}(t) &= a_{\mathrm{D},S_1} S_1(t) + a_{\mathrm{D},T} T(t) \\
f_{\mathrm{E}}(t) &= a_{\mathrm{E},S_1} S_1(t) + a_{\mathrm{E},g} g(t)
\end{aligned}
\tag{S4}
$$

where the following considerations were made. The sideband at zero delay is expected to produce a negative cross-correlation signal for band B and a positive cross-correlation signal for bands SB and E. The triplet state T primarily contributes to bands C and D, where it becomes dominant at delays exceeding 500 fs. The singlet state S_1 is expected to contribute to bands C, D, and E, while S_2 mainly affects band A. An additional contribution for band A is considered, proportional through the constant $a_{\mathrm{A,erf}}$ to the error function:

$$
\mathrm{erf}(t, t_0, \sigma) = \int_{-\infty}^{t} g(t, t_0, \sigma) dt \tag{S5}
$$

The inclusion of this term is justified by the fact that the signal associated with band A does not average to zero after the S_2 decays, which, as discussed later, can be associated with the presence of a hot ground state (HGS) long-lived population signature in this region. Note that the contribution of HGS was not directly taken into account by the model of Eq. (S3).

The fitting procedure was carried out using the `minimize` function from the `scipy.optimize` library, employing the Nelder-Mead optimization algorithm. The chi-squared error function to minimize was defined as:

$$\chi^2 = \sum_{j}^{N_j} \sum_{t}^{N_t} \left(\frac{\bar{f}_j(t) - f_j(t; \{p_k\})}{\sigma_{j,t}} \right)^2 \tag{S6}$$

where the sum over t runs and over the $N_t = 38$ temporal bins, $\sigma_{j,t}$ represents the standard error for band j at time t (shown as error bars in Fig. S10), $\bar{f}_j(t)$ is the measured yield for band j at time t (shown as dots in Fig. S10), and $f_j(t)$ is the function modeling band j at time t. The function $f_j(t)$ is parametrized by the set of $N_k = 15$ parameters p_k listed in Table S3, which include t_0, σ, t_1, t_2, and the set of amplitudes $\{a_{j,i}\}$ specific to the j-th band, as defined in Eqs. (S4). The starting parameters were initialized to physically reasonable values and constrained to be positive. The optimization process terminated successfully with the reduced chi-squared value $\chi_r^2 = \chi^2/(N_t \cdot N_j - N_k) = 0.9$, which, being very close to one, indicates a very good agreement between the model and the experimental data. The errors in the retrieved parameters were estimated by calculating the numerical Hessian matrix of the error function. The inverse of the Hessian matrix of the error function provides an estimate of the parameter covariance matrix, from which the standard deviations of the parameters are extracted. The formula for the error of each parameter, Δp_k, is:

$$\Delta p_k = \sqrt{(\mathrm{Cov}(p_k, p_k))} = \sqrt{((2\mathbf{H}^{-1})_{kk})} \ , \tag{S7}$$

where $\mathbf{H}^{-1}$ is the inverse of the Hessian matrix, and $(\mathbf{H}^{-1})_{kk}$ represents its diagonal elements, which are equal to half the variances of the model parameters. These errors reflect the uncertainty associated with each of the fit parameters.

To better understand the impact of these errors on the time-dependent contributions $f_j(t)$, we propagated the retrieved uncertainties through the model. This was achieved numerically by generating 500 parameter samples, each drawn from a Gaussian distribution with means and standard deviations corresponding to the values listed in Table S3. The resulting uncertainty plots for each $f_j(t)$ are shown in Fig. S11 as gray shaded scatter plots, and provide a visual representation of the confidence intervals associated with the fitted

contributions.

As it is possible to observe from Fig. S11, the uncertainty associated with the model generally overlaps with the experimental uncertainties, except in a few specific regions that will be addressed later. This visual agreement is consistent with the reduced chi-squared value being close to one, indicating a good fit between the model and the experimental data and further confirming the validity of the retrieved mean values and standard errors reported in Table S3.

One remarkable result is the very low value of t_1 obtained from the fit, which is $17 \pm 4\,\mathrm{fs}$, despite the cross-correlation between the pump and probe pulses being approximately 81 fs. At first glance, this result may seem surprising. The key to interpret this result lies in the fact that the model incorporates the sideband signal, which is proportional to the cross-correlation function and represents the instrument response function (IRF) of the system. Since the IRF is known, the experimental data can, in principle, be deconvolved from the IRF. This allows for a resolution that surpasses the FWHM of the IRF. Such an approach is well documented in the literature, where fitting techniques or deconvolution techniques combined with a knowledge of the IRF have been shown to achieve significantly higher temporal resolution than the nominal instrumental limit.[S32–S34]

In our specific case, the high accuracy achieved for the t_1 parameter arises from two key factors. First, we have a precise knowledge of the IRF, enabled by the direct observation of the sideband signal and the low signal-to-noise ratio in this region. Second, we can directly observe the rise time of the S_1 state from band D, which is not accessible in conventional visible pump-probe spectroscopy. As we will show soon, these two pieces of information alone are sufficient to determine the value of t_1, which means that the decay observed in band A, which is noisier and overlaps with the HGS, has a smaller influence on the retrieval of t_1. The model further refines this result by enforcing the condition that the decay time of band A and the rise time of band D are the same.

To visually illustrate this concept, Fig. S12(a) shows a comparison between $\mathrm{erf}(t)$ (blue

shaded dots) and $\hat{f}_D(t) = f_D(t)/a_{D,S_1}$ (orange shaded dots), both obtained from 500 samples generated using the mean and standard errors listed in Table S3. In the regime where $t_2 \gg t_1$, which applies to our case, the shift between the rise times of these two functions at half-maximum provides already a good estimate of t_1. As shown in the figure, this shift is clearly distinguishable and emerges above the model error. To quantify this shift, we computed the cross-difference (sample by sample) of the delays within the amplitude range of 0.4 to 0.6, as indicated by the red shaded dots in Fig. S12(b). The mean and standard deviation of these differences, represented by the red error bar, yields a value of 17.28 ± 4.18 fs. This result is consistent with the value obtained from the model, shown by the purple error bar in the same subplot. To exclude the contribution of the decay to the triplet state, we applied the same procedure by directly comparing erf(t) with the solution of the differential equation $d\hat{S}_1(t)/dt = S_2/t_2$, which represents the rising contribution of S_1 or, equivalently, the solution for S_1 when $t_2 \to \infty$. The S_1 obtained from the same 500 parameter samples is shown by black shaded dots in Fig. S12(a), which only start do deviate from $\hat{f}_D(t)$ in the region of $t > 60$ fs. The difference of the delays in the amplitude range of 0.4 to 0.6 is indicated by the black shaded dots in Fig. S12(c). The mean and standard deviation of these differences, represented by the black error bar, yield a value of 16.15 ± 4 fs. This result is slightly lower than the one obtained from the model (shown in purple in Fig. S12(c)) but still within the error margin. Nevertheless, the higher t_1 obtained from the model could be ascribed to an effect of band A, which slightly increases its value compared to the one obtained when only band D is considered.

Despite the goodness of the fit, some clear disagreements were observed, especially for the curves in panels A, C, D and E. These deviations are highlighted by the up and down arrows in Fig. S10, which indicate the direction of the offset between the experimental data and the fit result. The deviations show a periodic behavior and may have two different origins.

The energy regions A and C are close to the main ground state O8 and O7 peaks. The population of a HGS has been revealed by the scaled subtraction procedure (see main text

and section S5). The population of an HGS with binding energy oscillating around band B affects the signal around neighboring energy bands A and C. This is seen as an anti-correlated signal, A at lower kinetic energy and C at higher kinetic energy with respect to B (see arrow directions in Figs. S10 A and C). In principle, the population transfer to the HGS should be included in the model of Eq. S4. However, a clear formation time for the HGS could not be identified, while its presence, as shown later, is revealed by the Fourier analysis of these oscillations. For bands D and E, fast oscillations were attributed to the modulation of a normal mode in the excited S_1 state, and the observed deviations with respect to the fit are indicated by the arrows in the corresponding panels (see Fig. S10).

Taking these considerations into account, we estimated the period, phase, and amplitudes of the main oscillations observed in ranges A, C, D, and E of Fig. S10 using Fourier analysis. The experimental data for these bands are shown again as black dots in Figs. S13(a-d), corresponding to panels *a*, *b*, *c*, and *d*, respectively. The residuals, obtained by subtracting the previously obtained fit from the experimental data, are displayed as green dots in the same panels.

To isolate the region of positive delays, the residuals were multiplied by the error function (see Eq. (S5)). Subsequently, a Fourier Transform (FT) was performed, and the resulting power spectrum is presented in Figs. S13(e-h). The spectrum was fitted with a series of Gaussian modes, represented by shaded areas, whose central frequencies are marked by vertical lines with heights proportional to the mode power. The most relevant modes are highlighted in green: the oscillations in the time domain associated with these modes, after multiplication by the error function (Eq. (S5)), are represented by the green line in Figs. S13(a-d).

Starting from band C, we detect two main frequency components at 114.8 cm^{-1} (290.5 fs) and at 305.7 cm^{-1} (109.1 fs) (see Fig. S13(f)). The low frequency mode produces the oscillatory behaviour represented by a green line in Fig. S9(b), and is responsible for the disagreement observed in Fig. S6 (panel C). Also for band A we observe two main modes, very close in frequency to the ones detected for band C, the lower one being at 114.6 cm^{-1}

(291.1 fs) and the higher at 332.3 cm^{-1} (100.4 fs). The low frequency mode, the most intense, produces the oscillatory behaviour represented by the green solid line in Fig. S13(a). The two oscillatory signals shown in Figs. S13(a) and (b) are approximately out of phase ($\Delta\Phi \approx 1.3\pi$), as anticipated, confirming that they can be associated with the HGS binding energy oscillations around band B, which influence the signals in adjacent bands A and C.

For band D, two intense modes at 166.3 cm^{-1} (200.6 fs) and 227 cm^{-1} (146.9 fs) produce a beating with a central frequency of 198.1 cm^{-1} (168.4 fs), shown as a green line in Fig. S13(c). For band E, a single dominant mode at 292.7 cm^{-1} (113.9 fs) generates the oscillatory behaviour depicted as a green solid line in Fig. S13(d). The shorter period of oscillation for band E compared to D is in good agreement with our theoretical calculations (see section S10). Additionally, at around 200 fs the oscillations of D and E are in antiphase, as predicted by the theory.

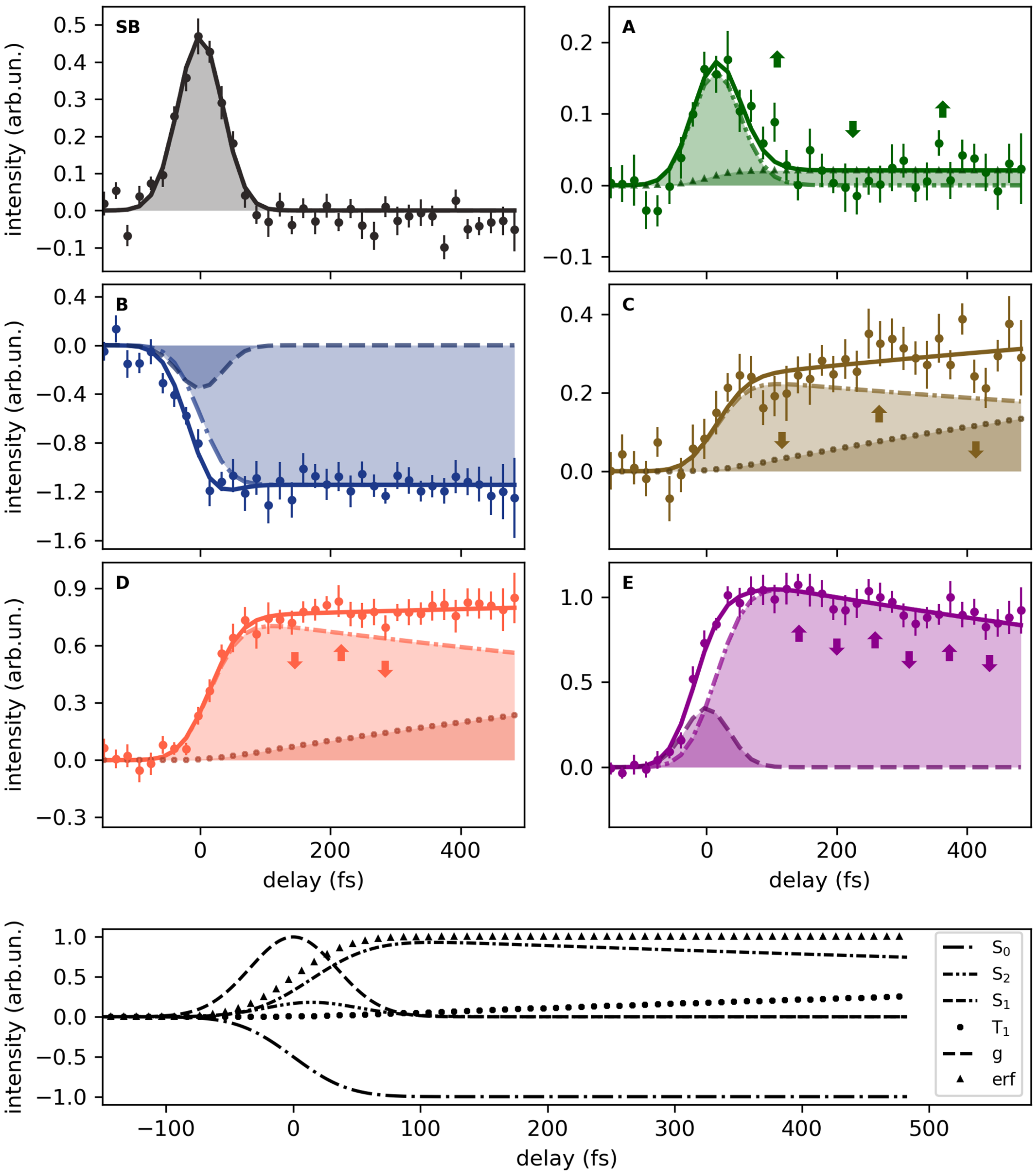

Figure S10: Experimental pump-probe differential yield for the six energy ranges SB (side-band), A, B, C, D, and E. The data points and standard errors are shown as dots and error bars, respectively. The solid lines represent the results of the fit to the data. For energy ranges A, B, C, D, and E, where two contributions are considered (see Eq. S4), both contributions, scaled by the fitted amplitude, are shown. The line styles of these contributions are defined in the legend of the bottom panel. Additionally, the best-fit solution to the differential equation for each state is plotted in the bottom panel, following the same line style coding. Up and down arrows in panels A, C, D and E indicate the observed clear deviations of the experimental data from the fit.

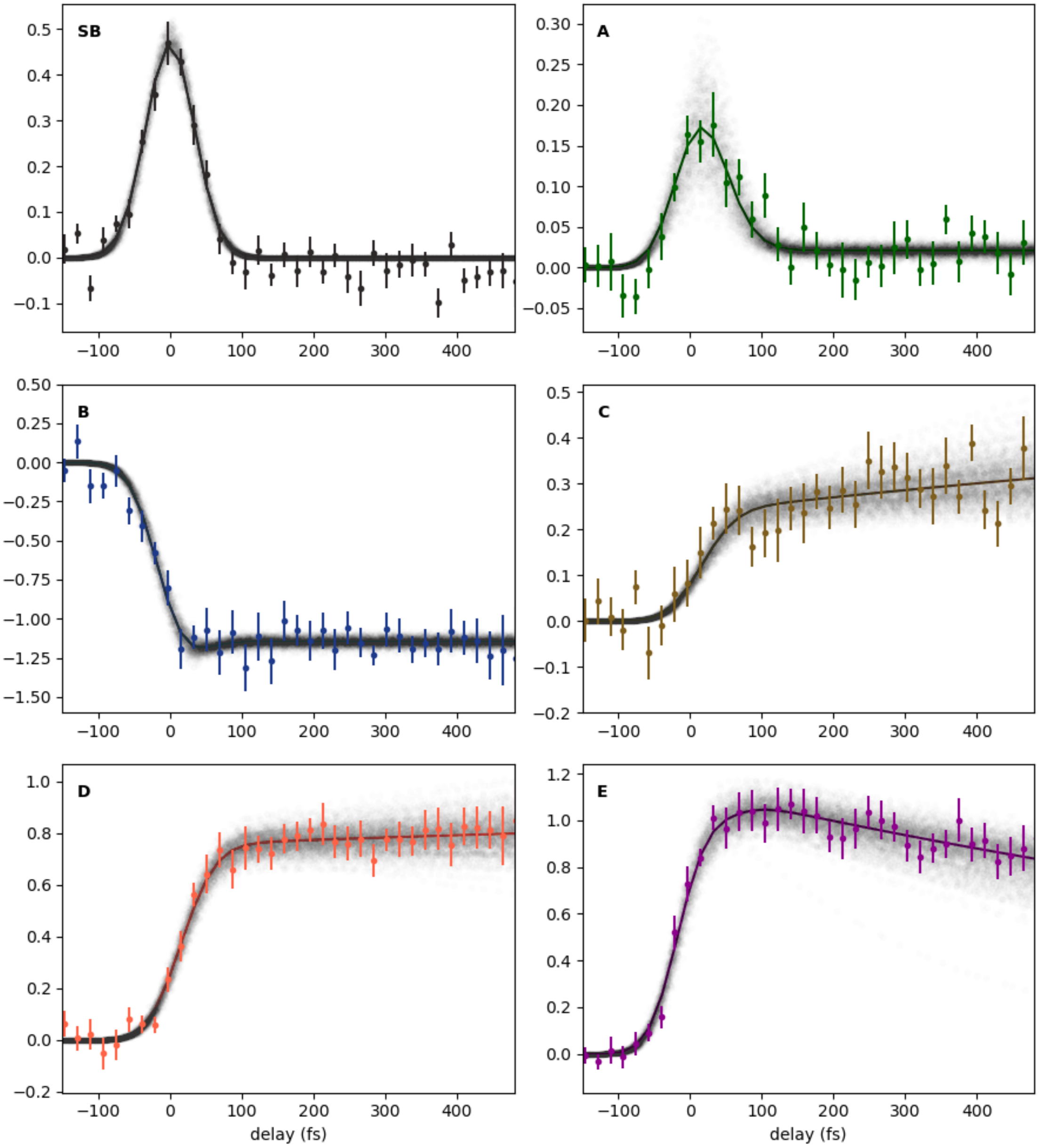

Figure S11: Experimental pump-probe differential yield for the six energy ranges SB (sideband), A, B, C, D, and E. The data points and standard errors are shown as dots and error bars, respectively. The solid lines represent the results of the fit to the data. The gray shaded scatter dots represent the values obtained from the 500 parameter samples, each drawn from a Gaussian distribution with means and standard deviations corresponding to the values listed in Table S3, and propagating these sample through the model.

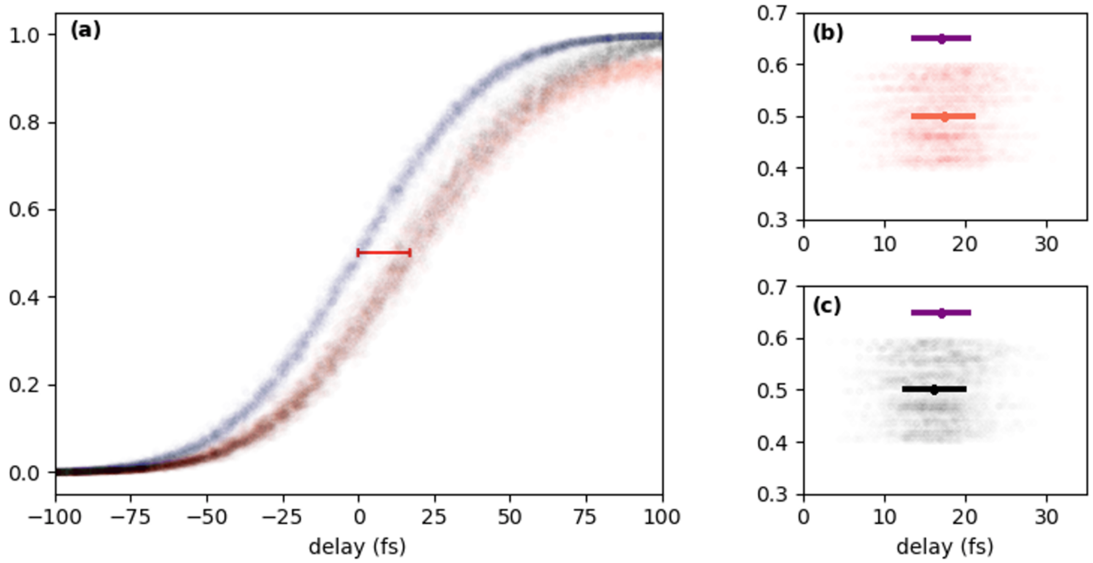

Figure S12: (a) Comparison of $\mathrm{erf}(t)$ (blue shaded dots) and $\hat{f}_D(t) = f_D(t)/a_{D,S_1}$ (orange shaded dots), obtained from 500 samples generated using the mean and standard errors listed in Table S3. The shift between the rise times of these two functions at half-maximum provides an estimate of t_1. The horizontal red bar corresponds to 17 fs. Also shown is $\hat{S}_1(t)$ (black shaded dots) for the same 500 samples, which is the rising contribution of S_1 ($t_2 \to \infty$). (b) Delays in the amplitude range of 0.4 to 0.6 of $\hat{f}_D(t)$ with respect to $\mathrm{erf}(t)$ (red shaded dots). The mean and standard deviation is represented by the red error bar. (c) Delays in the amplitude range of 0.4 to 0.6 of $\hat{S}_{1D}(t)$ with respect to $\mathrm{erf}(t)$ (black shaded dots). The mean and standard deviation is represented by the black error bar. In (b) and (c), the purple error bar indicates the value obtained from the model for comparison.

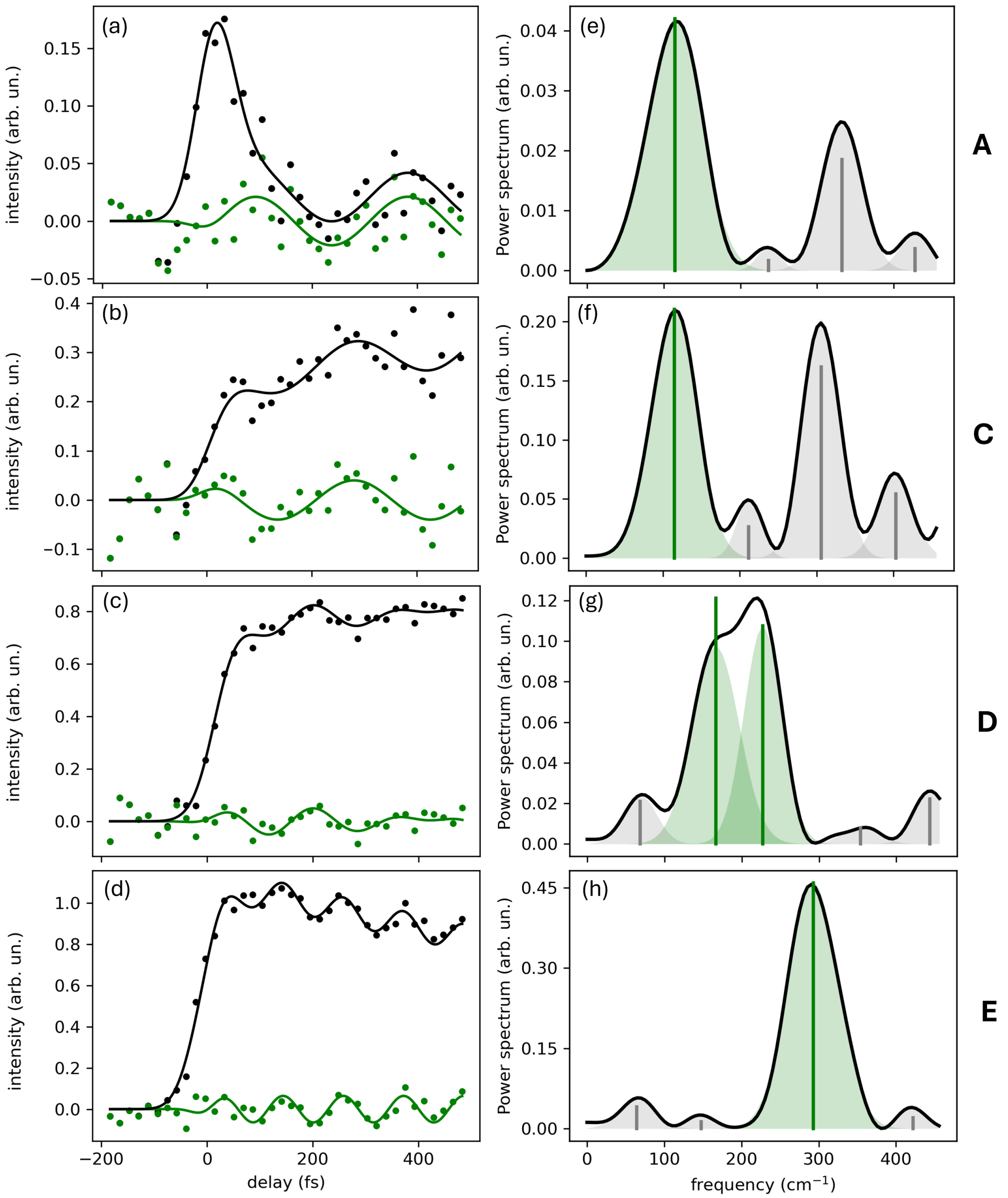

Figure S13: Panels (a-d) show the experimental data (black dots) for the energy bands corresponding to Fig. S6 panels A, C, D, and E. The green dots represent residuals obtained by subtracting the fit from the experimental data. The oscillations retrieved via Fourier analysis are shown as green lines, while the black line represents the sum of the fit and the retrieved oscillation. Panels (e-h) display the power spectrum of the residuals (black), with shaded areas representing the Gaussian modes identified by the multi-Gaussian fit. Each mode's central frequency is marked by a vertical line proportional to its power. Modes contributing to the green oscillations in panels (a-d) are highlighted in green.

S7 Direct relaxation pathway of uracil.

Figure S14 demonstrates that the trajectory remains in the diabatic $\pi\pi^*$ state and undergoes a direct $S_2(\pi\pi^*) \rightarrow S_1(\pi\pi^*) \rightarrow S_0$ deactivation pathway by internal conversion to the ground state *via* the ethylenic type S_1/S_0 CoIn.

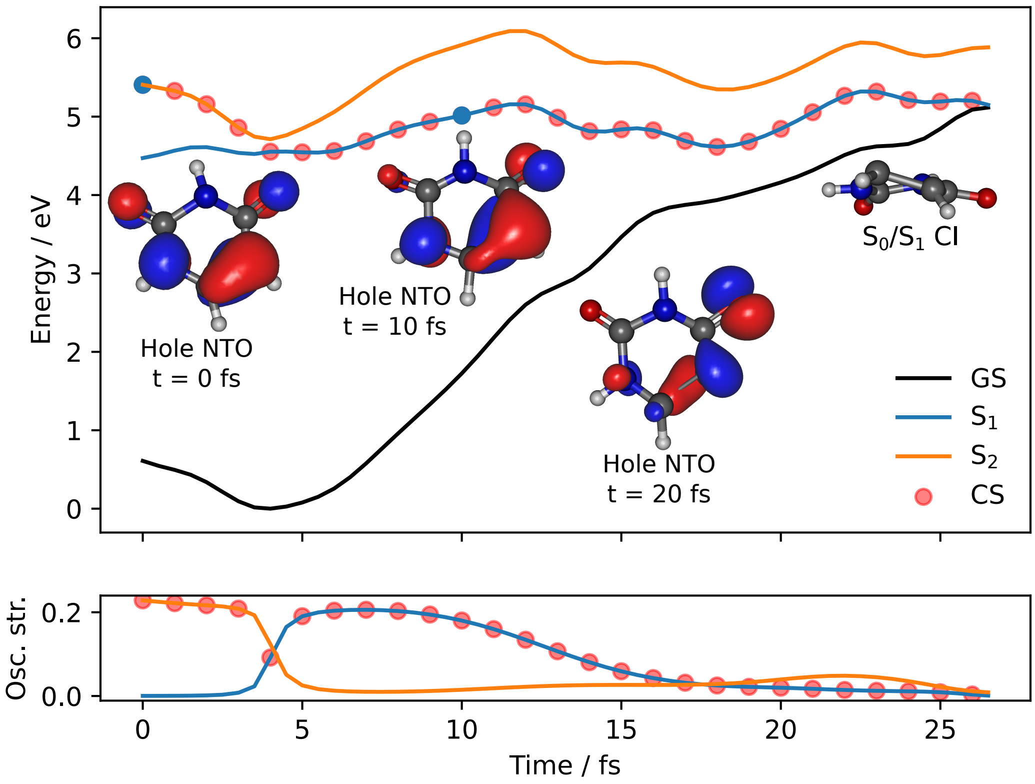

Figure S14: Top: time evolution of the potential energies of the two lowest singlet excited states. Orange: S_2. Blue: S_1. Black: ground state, S_0. The pink dots indicate the populated current state (CS) at a given time. The time steps for the hole NTOs (colored as blue dots) are also depicted in order to indicate that the currently populated states remain the same and do not change to the 'n' orbital. Bottom: the oscillator strengths of the S_2 and S_1 states. The CoIn between the initially populated $S_2(\pi\pi^*)$ and the $S_1(n\pi^*)$ states is encountered at around 5 fs. Analysis of the time evolution of the oscillator strength indicates that at the $S_2(\pi\pi^*)/S_1(n\pi^*)$ CoIn the system remains in the diabatic $\pi\pi^*$ state. The CoIn between the $S_1(\pi\pi^*)/S_0$ states is reached at around 25 fs.

S8 Calculated O 1s difference spectra.

Figure S15 shows the shake-up signal above the 546 eV region in the spectra for a selected SH trajectory which undergoes internal conversion to $S_1(n\pi^*)$ state.

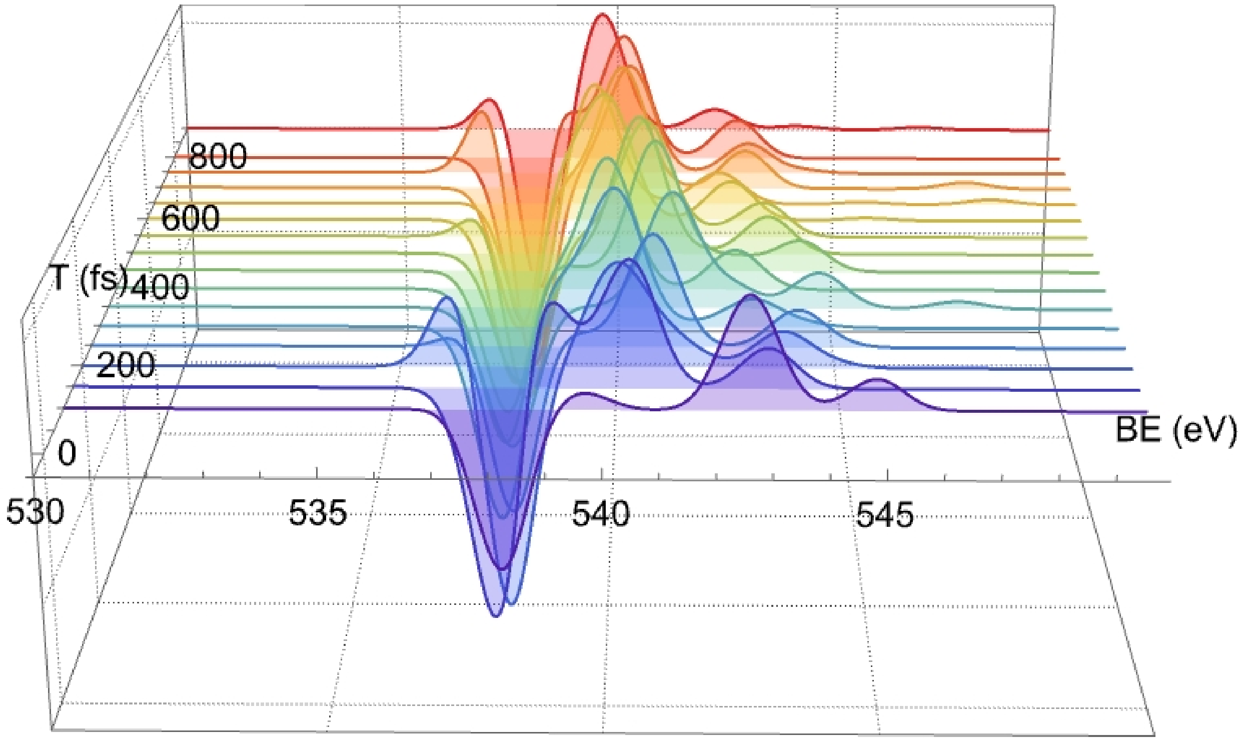

Figure S15: Calculated O 1s difference spectra (excited state minus ground state) for a selected SH trajectory of uracil undergoing internal conversion to the dark $S_1(n\pi^*)$ state. The spectra were calculated from 100 to 900 fs in 50 fs steps.

.

S9 Depletion of the N 1s signal.

Figure S16 shows the intensity of the sidebands of the N 1s signal and of the depletion of the main line, integrated over the binding energies of the respective peaks. It can be seen that adding the intensity of the two sidebands to the depletion signal cancels out the anomalous recovery of the depletion.

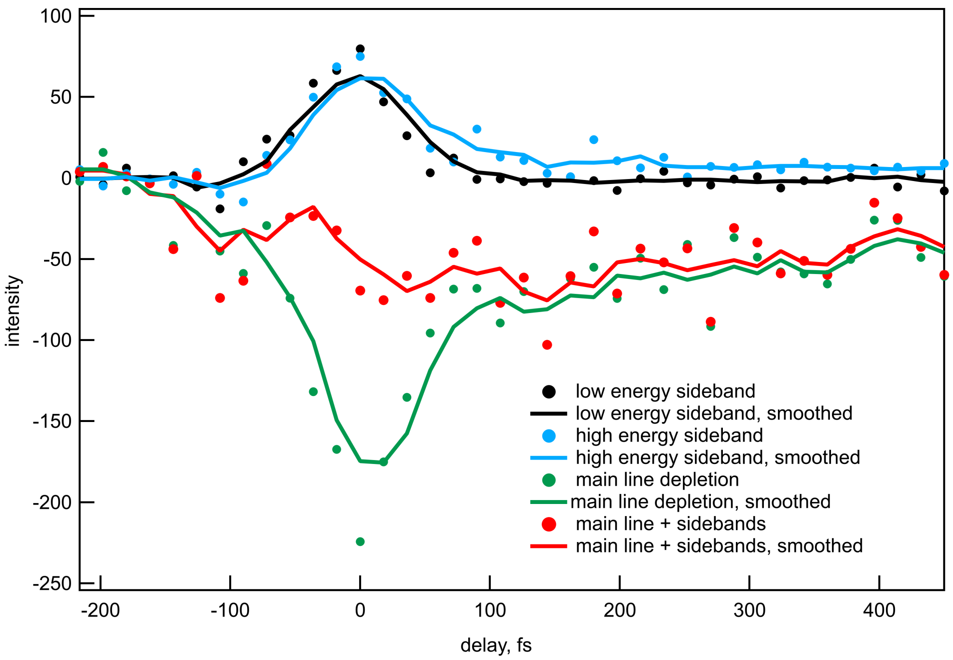

Figure S16: Intensities of the N 1s features in Fig. 3(b) (main text), integrated over binding energy. Top curves (blue, black): lower and higher energy sidebands. Bottom curve (green): depletion of main line. Intermediate curve (red): sideband-corrected depletion of the main line (sum of sideband and depletion curves.)

S10 Calculated average bond lengths.

To investigate the origin of oscillations in the O 1s signal intensities, we first analyzed the system in terms of ground-state normal modes. After minimizing the RMSD (root-mean-square deviation) between the excited and reference ground-state geometries, we projected all geometries from all SH trajectories onto the MP2/cc-pVDZ normal mode displacement vectors. The averaged normal mode displacements suggested potential candidates for the observed low-frequency oscillations. However, the complexity of the system and the significant displacement from the ground-state equilibrium following UV excitation make a definitive assignment challenging.

To further explore these oscillations, we computed the average lengths of the C5=C6 and C4=O8 bonds, which exhibit substantial elongation within the first ~ 20 fs of SH dynamics in the $S_2(\pi\pi^*)$ state. Specifically, the C4=O8 bond extends from 1.26 Å to 1.48 Å, while the C5=C6 bond increases from 1.40 Å to 1.63 Å. We analyzed trajectories in the $S_2(\pi\pi^*)$ and $S_1(n\pi^*)$ states separately. As shown in Fig.S17, oscillations are more pronounced in the $\pi\pi^*$ state due to the sudden and simultaneous elongation of both bonds upon $\pi \to \pi^*$ excitation. Oscillations in the $n\pi^*$ state are weaker, as this state is populated more gradually at later times, allowing partial vibrational energy redistribution into other modes. Nevertheless they remain clearly visible. Our calculations indicate that the C5=C6 and C4=O8 bond distances oscillate with periods of approximately 100 fs and 80 fs, respectively, in good agreement with the observed signal modulations. Based on this, we interpret the periodic intensity variations in the S_2 and S_1 states as a vibronic effect.

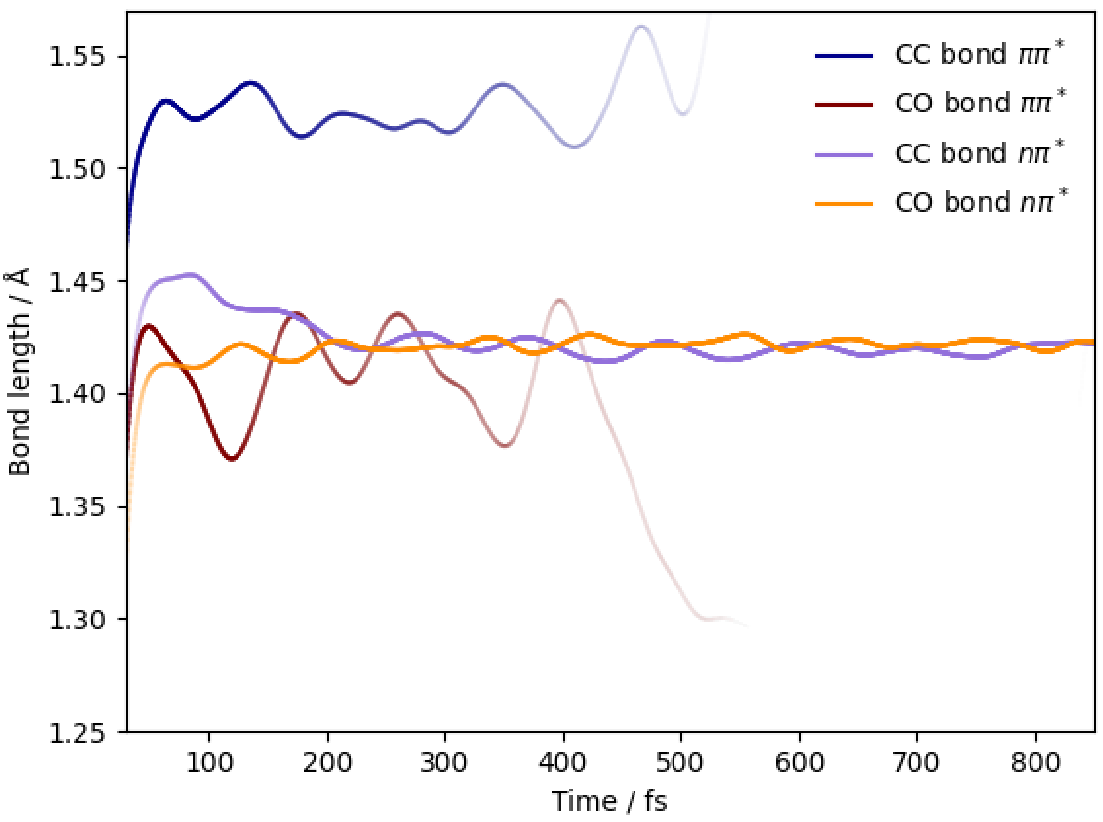

Figure S17: Average bond distances of C5=C6 (CC) and C4=O8 (CO) calculated over a set of trajectories, and convoluted with a Gaussian function of 40 fs FWHM for the ($\pi\pi^*$) and ($n\pi^*$) states of uracil. The opacity of each line matches the number of trajectories which are in the corresponding state. The fading of the upper and lower lines is due to the depopulation/population of the S_2/S_1 states, respectively.

S11 Geometries of the relevant minima and conical intersections.

Figure S18 illustrates the five relevant structures of uracil: S_0 (i) and S_2 (ii) minima, S_2/S_1 (iii) minimum energy CoIn (MECoIn), S_1/S_0 (iv) CoIn, S_1 (v) minimum, respectively of uracil. The structures were optimized at the SCS-ADC(2)/aug-cc-pVDZ level of theory. Going from the S_0 to the S_1 minimum, the main geometrical changes observed are the elongation of the C4=O8 and C5=C6 double bonds, and the simultaneous shortening of the C4-C5 bond. There are two important CoIns in uracil that are characterized by a strong out-of-plane distortion at the C5 carbon atom (see Figs. S18(iii) and (iv)). The present SH calculations indicate that the ultrafast initial change of uracil geometry (a combination of bond stretching, bending, torsion, etc.) takes place within 20-30 fs and after that, the molecule starts to vibrate around the new equilibrium structure.

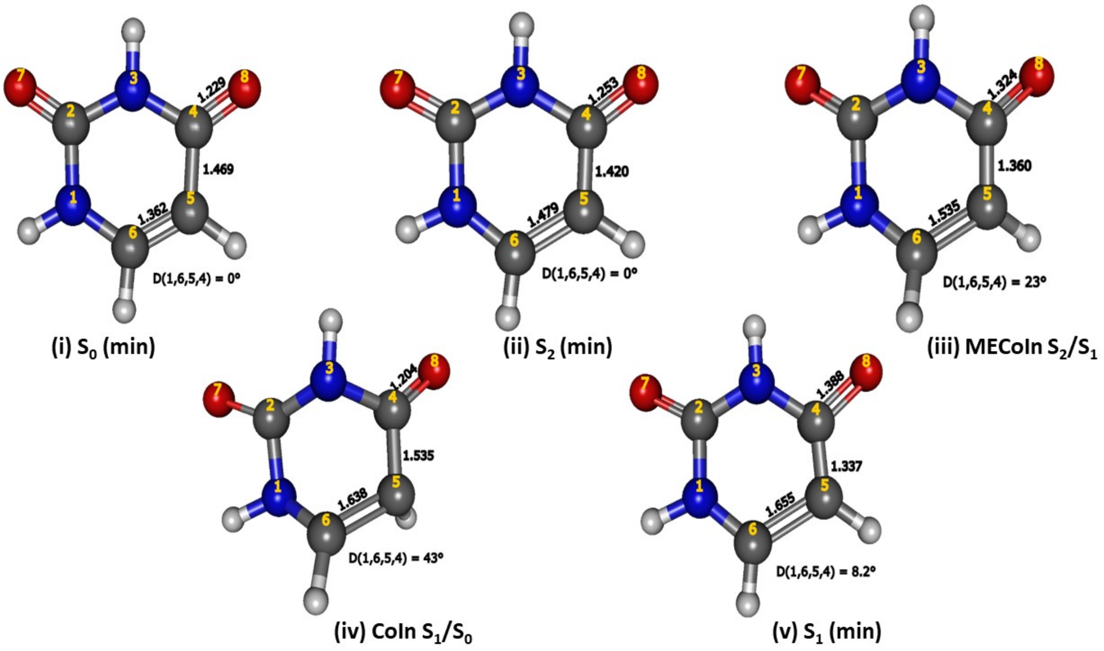

Figure S18: Relevant minima (i, ii and v) and conical intersections (iii and iv) for the photophysics of uracil. Selected bond lengths (labeled in Å) are presented only where significant structural changes take place.

S12 Time-resolved C 1s spectra of uracil.

The theoretical and experimental C K-edge time-resolved photoelectron spectra of UV-excited uracil are presented in Figure S19. Calculated C 1s spectra (see Fig. S19(a)) of the electronic ground (S_0) and the lowest energy excited (S_1) states were computed for the appropriate minimum energy structure geometries (see Fig. S18(i and v)).

Uracil has four carbon atoms in its structure, hence the observed negative signal along the whole time window at 291 eV, 292.8 eV, 294.4 eV, and 295.4 eV is assigned to the ground state depletion of C5, C6, C4 and C2, respectively[S18] (see Fig. S19(b)). The intensity at low BE (from 285 to 290 eV) around $t = 0$ is due to the four sideband signals, and is consistent with the number of carbon atoms.

The negative charge accumulation on the C6, C5 and C4 atoms is responsible for the initial shift for these carbon atoms relative to the GS spectrum immediately after photoexcitation (see Table S2). However, the C 1s TR-XPS spectra measured here turned out to be more sensitive to the structural deformation than to the partial charges. According to Matsika et al.,[S35] internal conversion from $S_2 \rightarrow S_1$ occurs through the conical intersection caused by bond elongation in a planar geometry (see Fig. S18(iii)). On the other hand, the sub-30 fs relaxation pathway from $S_2 \rightarrow S_0$ is induced by a twist of the ethylenic C5=C6 double bond and a strong out-of-plane distortion of the uracil ring (see Fig. S18 (iv))[S2,S35–S38]. Hence, the experimentally observed and computed spectrum for the S_1 state asymmetric signal at around 292 eV (see Fig. S19(a, b), range A) is assigned to the different deactivation channels (direct and indirect) induced by deformations in the uracil structure, with the biggest changes happening in the vicinity of the carbon C5 atom.

Note that C K-edge simulations as a function of time were outside the scope of the present work due to the limited statistics and the higher computational cost compared to the O and N K-edges.

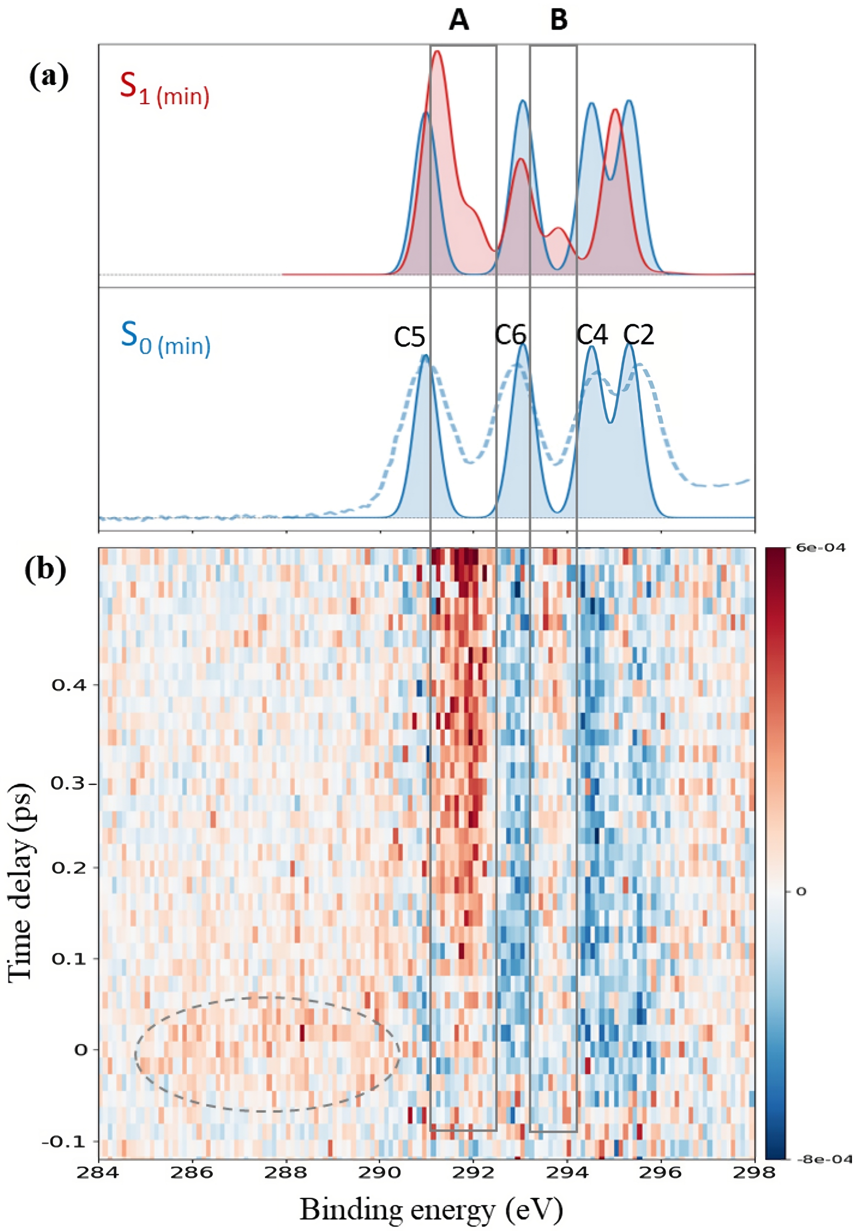

Figure S19: (a) Calculated C 1s spectra of the S_0 ground state (blue) and the excited S_1 (red) state of uracil computed at their minimum energy geometries. Theoretical spectra are shifted by 2 eV to lower binding energy. The blue dashed line represents the experimentally measured GS spectrum of uracil. (b) Two-dimensional false color map of the C 1s subtraction spectra (UV-on minus UV-off) as a function of binding energy and of time delay (red: positive signal, blue: negative signal). Gray dashed circle indicates four sidebands (see text). Energy ranges (eV): (A) 291.0 - 292.5 and (B) 293.0 - 294.3.

References

(S1) Tully, J. C. Molecular dynamics with electronic transitions. *J. Chem. Phys.* **1990**, *93*, 1061–1071.

(S2) Milovanović, B.; Novak, J.; Etinski, M.; Domcke, W.; Došlić, N. Simulation of UV absorption spectra and relaxation dynamics of uracil and uracil–water clusters. *Phys. Chem. Chem. Phys.* **2021**, *23*, 2594–2604.

(S3) Grimme, S. Improved second-order Møller–Plesset perturbation theory by separate scaling of parallel- and antiparallel-spin pair correlation energies. *J. Chem. Phys.* **2003**, *118*, 9095–9102.

(S4) Hellweg, A.; Grün, S. A.; Hättig, C. Benchmarking the performance of spin-component scaled CC2 in ground and electronically excited states. *Phys. Chem. Chem. Phys.* **2008**, *10*, 4119–4127.

(S5) Schirmer, J. Beyond the random-phase approximation: A new approximation scheme for the polarization propagator. *Phys. Rev. A* **1982**, *26*, 2395.

(S6) Dreuw, A.; Wormit, M. The algebraic diagrammatic construction scheme for the polarization propagator for the calculation of excited states. *Wiley Interdiscip. Rev.: Comput. Mol. Sci.* **2015**, *5*, 82–95.

(S7) Sapunar, M.; Piteša, T.; Davidović, D.; Došlić, N. Highly efficient algorithms for CIS type excited state wave function overlaps. *J. Chem. Theory Comput.* **2019**, *15*, 3461–3469.

(S8) TURBOMOLE V7.0 2015, a development of University of Karlsruhe and Forschungszentrum Karlsruhe GmbH, 1989-2007, TURBOMOLE GmbH, since 2007; available from `http://www.turbomole.com`.

(S9) Tajti, A.; Tulipan, L.; Szalay, P. Accuracy of Spin-Component Scaled ADC(2) Excitation Energies and Potential Energy Surfaces. *J. Chem. Theory Comput.* **2020**, *16*, 468–474.

(S10) Mai, S.; Avagliano, D.; Heindl, M.; Marquetand, P.; Menger, M.; Oppel, M.; Plasser, F.; Polonius, S.; Ruckenbauer, M.; Shu, Y. et al. SHARC3.0: Surface Hopping Including Arbitrary Couplings – Program Package for Non-Adiabatic Dynamics, https://sharc-md.org/. 2023.

(S11) Mai, S.; Plasser, F.; Pabst, M.; Neese, F.; Köhn, A.; González, L. Surface hopping dynamics including intersystem crossing using the algebraic diagrammatic construction method. *J. Chem. Phys.* **2017**, *147*, 184109.

(S12) Neese, F. Software update: The ORCA program system—Version 5.0 . *Wiley Interdiscip. Rev. Comput. Mol. Sci.* **2022**, *12*, e1606.

(S13) Granucci, G.; Persico, M.; Toniolo, A. Direct semiclassical simulation of photochemical processes with semiempirical wave functions. *J. Chem. Phys.* **2001**, *114*, 10608–10615.

(S14) Granucci, G.; Persico, M. Critical appraisal of the fewest switches algorithm for surface hopping. *J. Chem. Phys.* **2007**, *126*, 134114.

(S15) Mayer, D.; Lever, F.; Picconi, D.; Metje, J.; Alisauskas, S.; Calegari, F.; Düsterer, S.; Ehlert, C.; Feifel, R.; Niebuhr, M. et al. Following excited-state chemical shifts in molecular ultrafast x-ray photoelectron spectroscopy. *Nat. Commun.* **2022**, *13*, 198.

(S16) Toffoli, D.; Coriani, S.; Stener, M.; Decleva, P. Tiresia: A code for molecular electronic continuum states and photoionization. *Comput. Phys. Commun.* **2024**, *297*, 109038.

(S17) van Leeuwen, R.; Baerends, E. J. Exchange-correlation potential with correct asymptotic behavior. *Phys. Rev. A* **1994**, *49*, 2421–2431.

(S18) Feyer, V.; Plekan, O.; Richter, R.; Coreno, M.; Vall-Llosera, G.; Prince, K. C.; Trofimov, A. B.; Zaytseva, I. L.; Moskovskaya, T. E.; Gromov, E. V. et al. Tautomerism in cytosine and uracil: An experimental and theoretical core level spectroscopic study. *J. Phys. Chem. A* **2009**, *113*, 5736–5742.

(S19) Mazza, T.; Baumann, T. M.; Boll, R.; De Fanis, A.; Grychtol, P.; Ilchen, M.; Montaño, J.; Music, V.; Ovcharenko, Y.; Rennhack, N. et al. The beam transport system for the Small Quantum Systems instrument at the European XFEL: optical layout and first commissioning results. *J. Synchrotron Radiat.* **2023**, *30*, 457–467.

(S20) Sinn, H.; et al. X-Ray Optics and Beam Transport, Technical Design Report [10.3204/XFEL.EU/TR-2012-006]. *XFEL. EU. Technical Report* **2012**, 164.

(S21) Serkez, S.; Gorobtsov, O.; Rivas, D. E.; Meyer, M.; Sobko, B.; Gerasimova, N.; Kujala, N.; Geloni, G. Wigner distribution of self-amplified spontaneous emission free-electron laser pulses and extracting its autocorrelation. *J. Synchrotron Radiat.* **2021**, *28*, 3–17.

(S22) Serkez, S.; Gorobtsov, O.; Sobko, B.; Gerasimova, N.; Geloni, G. ROSA: Reconstruction of Spectrogram Autocorrelation for Self-Amplified Spontaneous Emission Free-Electron Lasers. *arXiv preprint arXiv:1811.11446* **2018**, 1–19.

(S23) Gerasimova, N.; La Civita, D.; Samoylova, L.; Vannoni, M.; Villanueva, R.; Hickin, D.; Carley, R.; Gort, R.; Van Kuiken, B.; Miedema, P. et al. The soft X-ray monochromator at the SASE3 beamline of the European XFEL: from design to operation. *J. Synchrotron Radiat.* **2022**, *29*, 1299–1308.

(S24) Koch, A.; Risch, J.; Freund, W.; Maltezopoulos, T.; Planas, M.; Grünert, J. Operation of photon diagnostic imagers for beam commissioning at the European XFEL. *Synchrotron Radiat.* **2019**, *26*, 1489–1495.

(S25) Coreno, M.; Avaldi, L.; Camilloni, R.; Prince, K.; De Simone, M.; Karvonen, J.; Colle, R.; Simonucci, S. Measurement and ab initio calculation of the Ne photoabsorption spectrum in the region of the K edge. *Phys. Rev. A* **1999**, *59*, 2494–2497.

(S26) Pergament, M.; Palmer, G.; Kellert, M.; Kruse, K.; Wang, J.; Wissmann, L.; Wegner, U.; Emons, M.; Kane, D.; Priebe, G. et al. Versatile optical laser system for experiments at the European X-ray free-electron laser facility. *Opt. Express* **2016**, *24*, 29349–29359.

(S27) Grychtol, P.; Rivas, D. E.; Baumann, T. M.; Boll, R.; De Fanis, A.; Erk, B.; Ilchen, M.; Liu, J.; Mazza, T.; Montaño, J. et al. Timing and X-ray pulse characterization at the Small Quantum Systems instrument of the European X-ray Free Electron Laser. *Opt. Express* **2021**, *29*, 37429–37442.

(S28) McFarland, B.; Berrah, N.; Bostedt, C.; Bozek, J.; Bucksbaum, P.; Castagna, J.; Coffee, R.; Cryan, J.; Fang, L.; Farrell, J. et al. Experimental strategies for optical pump–soft x-ray probe experiments at the LCLS. *J. Phys. Conf. Ser.* **2014**, *488*, 012015.

(S29) Kruit, P.; Read, F. Magnetic field paralleliser for 2π electron-spectrometer and electron-image magnifier. *J. Phys. E: Sci. Instrum.* **1983**, *16*, 313–324.

(S30) Hikosaka, Y.; Sawa, M.; Soejima, K.; Shigemasa, E. A high-resolution magnetic bottle electron spectrometer and its application to a photoelectron–Auger electron coincidence measurement of the $L_{2,3}$ VV Auger decay in CS_2. *J. Electron Spectros. Relat. Phenomena* **2014**, *192*, 69–74.

(S31) Borne, K.; O'Neal, J. T.; Wang, J.; Isele, E.; Obaid, R.; Berrah, N.; Cheng, X.; Bucksbaum, P. H.; James, J.; Kamalov, A. et al. Design and performance of a magnetic bottle electron spectrometer for high-energy photoelectron spectroscopy. *Rev. Sci. Instrum.* **2024**, *95*, 125110.

(S32) Iagatti, A.; Doria, S.; Marcelli, A.; Angelini, N.; Notarantonio, S.; Paoletti, A. M.; Pennesi, G.; Rossi, G.; Zanotti, G.; Calogero, G. et al. Photophysical processes occurring in a Zn-phthalocyanine in ethanol solution and on TiO_2 nanostructures. *J.Phys. Chem. C* **2015**, *119*, 20256–20264.

(S33) Fazel, M.; Vallmitjana, A.; Scipioni, L.; Gratton, E.; Digman, M. A.; Pressé, S. Fluorescence lifetime: Beating the IRF and interpulse window. *Biophys. J.* **2023**, *122*, 672–683.

(S34) Mouton, N.; Sliwa, M.; Buntinx, G.; Ruckebusch, C. Deconvolution of femtosecond time-resolved spectroscopy data in multivariate curve resolution. Application to the characterization of ultrafast photo-induced intramolecular proton transfer. *J. Chemom.* **2010**, *24*, 424–433.

(S35) Matsika, S. Radiationless decay of excited states of uracil through conical intersections. *J. Phys. Chem. A* **2004**, *108*, 7584–7590.

(S36) Carbonniere, P.; Pouchan, C.; Improta, R. Intramolecular vibrational redistribution in the non-radiative excited state decay of uracil in the gas phase: an ab initio molecular dynamics study. *Phys. Chem. Chem. Phys.* **2015**, *17*, 11615–11626.

(S37) Richter, M.; Mai, S.; Marquetand, P.; González, L. Ultrafast intersystem crossing dynamics in uracil unravelled by ab initio molecular dynamics. *Phys. Chem. Chem. Phys.* **2014**, *16*, 24423–24436.

(S38) Miura, Y.; Yamamoto, Y.-i.; Karashima, S.; Orimo, N.; Hara, A.; Fukuoka, K.; Ishiyama, T.; Suzuki, T. Formation of Long-Lived Dark States during Electronic Relaxation of Pyrimidine Nucleobases Studied Using Extreme Ultraviolet Time-Resolved Photoelectron Spectroscopy. *J. Am. Chem. Soc.* **2023**, *145*, 3369–3381.
